# Thermodynamic Study of Amine-Based Deep Eutectic Solvents with EtOH

**DOI:** 10.1021/acs.jced.6c00157

**Published:** 2026-07-13

**Authors:** Fei Lin, Yusi Shen, Zhida Zuo, Linghong Lu, Xiaohua Lu, Yudan Zhu, Xiaoyan Ji

**Affiliations:** † State Key Laboratory of Materials-Oriented Chemical Engineering, 506240 Nanjing Tech University, Nanjing 210009, P. R. China; ‡ Division of Energy Science/Energy Engineering, Luleå University of Technology, 97187 Luleå, Sweden; § Suzhou Laboratory, Suzhou 215125, P. R. China

## Abstract

The densities and viscosities of the three amine-based deep eutectic solvents (DESs), i.e., [EmimCl]­[MEA], [BmimCl]­[MEA], and [HmimCl]­[MEA], mixed with ethanol (EtOH), were measured from 288.15 to 323.15 K, and their enthalpies of mixing were determined at 298.15 and 308.15 K. Negative excess molar volumes across the entire compositional range indicate significant volume contraction and enhanced molecular packing, and their temperature dependence suggests the dominance of packing effects over hydrogen-bonding. Viscosity deviations show a characteristic U-shaped composition dependence, reflecting disruption of the original DES hydrogen-bond network by EtOH. The enthalpy of mixing changes from positive to negative as the DES composition increases, revealing strengthened DES–EtOH interactions. The distinct composition-dependent trends observed for these three (DES + EtOH) systems indicate that variations in alkyl chain length modulate molecular packing and interaction strength, resulting in nonuniform ordering patterns across thermophysical properties. Compared to water, which strongly restructures the DES hydrogen-bond network and produces significant exothermic mixing effects, EtOH exerts a milder structural and thermodynamic impact. This work provides reliable thermodynamic data and mechanistic insight for the design and application of amine-based DESs.

## Graphical abstract

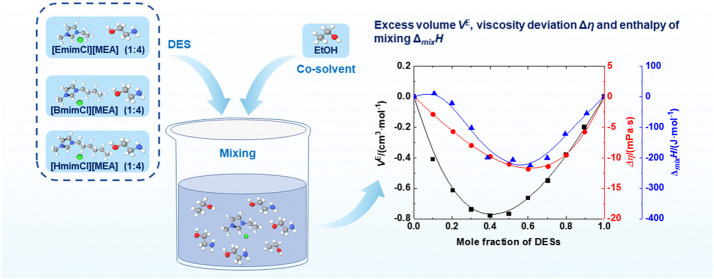

## 1 Introduction

The intensifying greenhouse effect and environmental challenges associated with carbon dioxide (CO_2_) emissions have raised significant global concern, prompting extensive research into CO_2_ capture technologies in recent years.^1^ Current CO_2_ capture approaches primarily include absorption,^2,3^ adsorption,^3,4^ membrane separation^5^ and related methods. Among these technologies, chemical absorption has been widely implemented due to its technological maturity, with amine-based absorbents being the most representative systems.^6,7^ However, traditional amine-based absorbents suffer from several inherent drawbacks, such as high energy demand for generation, severe corrosiveness, and considerable solvent volatility.^7,8^

Ionic liquids (ILs) have emerged as promising alternatives to traditional amine solvents because of their high CO_2_ solubilities, excellent thermal stability, and tunable chemical structures, which enable enhanced capture efficiency.^9^ Deep eutectic solvents (DESs) are often termed IL analogues owing to their high physicochemical similarity to ILs.^10^ DESs are typically synthesized by simple mixing of hydrogen bond acceptors (HBAs) and hydrogen bond donors (HBDs), offering advantages of low cost and straightforward preparation.^10,11^ Consequently, they have attracted considerable attention for industrial applications in recent years.^12^ In particular, amine-based DESs, i.e., amine compounds that act as HBDs, have emerged as highly efficient CO_2_ absorbents owing to their superior absorption capacity and selectivity compared with traditional DESs.^13,14^ Common amine-based HBDs include monoethanolamine (MEA), diethanolamine (DEA), diethylenetriamine (DETA), and ethylenediamine (EDA).^12,13^

A major challenge associated with amine-based DESs is their high viscosity, which significant influences mass transfer and energy use in the CO_2_ capture process.^13,15^ The introduction of cosolvents has demonstrated to be an effective strategy for viscosity reduction.^16–18^ Previous studies have shown that cosolvents (e.g., H_2_O) can significantly alter the microstructure and intermolecular interactions within DESs.^19–21^ Owing to its strong hydrogen-bonding (H-bonding) interactions and its ability to screen Coulombic interactions between ions, the cosolvent of H_2_O can disrupt the original hydrogen-bond (H-bond) network of DESs and promote the formation of new H-bonding interactions.^20,22–24^ Such structural rearrangement may induce pronounced changes in the internal microstructure and intermolecular interactions, potentially leading to the formation of ternary DES systems.^22^ The reduced viscosity of DES mixtures while largely preserving their DES-like structures may provide a feasible strategy for balancing CO_2_ absorption capacity with improved transport properties, which is crucial for solvent and process design. However, the current understanding remains insufficient to elucidate the behavior observed in the (amine-based DES + cosolvent) systems, particularly with respect to their structure–property relationships.^25^ Therefore, systematic investigations of key physicochemical properties are essential to advance both fundamental understanding and practical application of these systems.

Although the CO_2_ absorption performance of DESs based on imidazolium-based ILs as HBAs may not surpass that of some amine-based DESs,^26–29^ they remain valuable model systems because their physicochemical properties and intermolecular interactions are well documented.^24,28^ Meanwhile, MEA can be a desirable HBD due to its established role in CO_2_ capture and the presence of dual functional groups (−NH_2_ and −OH) to promote the formation of stable DES networks through strong H-bonding interactions.^28,29^ Accordingly, DESs composed of imidazolium chlorides and MEA provide suitable model systems for probing structure–property relationships in CO_2_ capture applications. From the HBA perspective, these systems can be regarded as IL-based DESs; however, since MEA is the primary HBD of interest and imidazolium ILs mainly serve as model components, they are referred to here as amine-based DESs. Compared with conventional choline chloride-based DESs, these amine-based DESs exhibit more complex H-bonding networks because the imidazolium cations and Cl^–^ anions interact with the HBD.^23,28,29^ They also form stronger and more structured H-bonding interactions than corresponding (IL + amine) mixtures.^28,29^ Previous studies have shown that the DES composed of [BmimCl] and MEA at a molar ratio of 1:4 exhibits the highest CO_2_ absorption capacity,^28^ and in our previous study, the densities, viscosities, and enthalpies of mixing of the three aqueous amine-based DESs, i.e., [EmimCl]­[MEA] (1:4), [BmimCl]­[MEA] (1:4), and [HmimCl]­[MEA] (1:4), were systematically studied, indicating the important role of the solvent polarity in governing the behavior of (DES + cosolvent) systems.^24^ Additionally, these physicochemical properties are essential for evaluating DES-based CO_2_ capture systems, because they provide key inputs for developing and validating thermodynamic and transport property models, enabling reliable correlation and prediction of DES performance in CO_2_ capture processes. As H_2_O represents an extreme case of a highly polar solvent, and its strong interactions may mask more general solvent effects, such as molecular packing contributions, and moderate polarity influences, it is important to extend the work on these DESs to their mixtures with other organic cosolvents.

To address this gap, in this work, ethanol (EtOH), with a lower dielectric constant compared to H_2_O,^30^ was selected as a representative organic cosolvent of [EmimCl]­[MEA] (1:4), [BmimCl]­[MEA] (1:4), and [HmimCl]­[MEA] (1:4). Owing to its moderate polarity and weaker H-bonding ability, EtOH is expected to exert a milder structural perturbation in the DES network, which enables differentiation between H-bond-driven structural reorganization and molecular packing effects. Based on this framework, these DESs were prepared at a fixed molar ratio of 1:4, and then mixed with EtOH. Densities and viscosities of these three (DES + EtOH) systems were measured over 288.15–323.15 at 5 K intervals. Excess volumes and viscosity deviations were further obtained to characterize nonideal behavior. Additionally, enthalpies of mixing were determined at 298.15 and 308.15 K across the full compositional range and analyzed, providing further insight into the thermodynamic nonideality in the amine-based (DES + EtOH) systems.

## 2 Experimental Section

### 2.1 Chemicals and Preparation of DESs

Detailed information on the chemicals used in this study is summarized in Table 1. To eliminate residual moisture of [EmimCl], [BmimCl], and [HmimCl], all were dried in a vacuum oven containing P_2_O_5_ at 343 K under a pressure of 0.01 bar for 4 days prior to use. MEA and EtOH were used as received without further purification. Specifically, EtOH was stored in sealed vials with molecular sieves to maintain its water content below 50 ppm. The dried [EmimCl], [BmimCl], and [HmimCl], together with MEA and EtOH, were subsequently transferred to a vacuum glovebox with ultralow water content (<0.01 ppm) for the preparation of the DESs and their mixtures with EtOH. 3 Å molecular sieves were activated by calcination at 623 K for 8 h and subsequently placed in sealed vials for the DES storage.

**Table 1:** Chemicals Used in This Work

Chemical name	Compound abbreviations	Molecular formula	CAS number	Molar mass (g·mol^–1^)	Purity (mass fraction)	Source
1-Ethyl-3-methylimidazolium chloride	[EmimCl]	C_6_H_11_ClN_2_	65039–09–0	146.62	0.99	Lanzhou Institute of Chemical Physics, Chinese Academy of Sciences
1-Butyl-3-methylimidazolium chloride	[BmimCl]	C_8_H_15_ClN_2_	79917–90–1	174.67	0.99	
1-Hexyl-3-methylimidazolium chloride	[HmimCl]	C_10_H_19_ClN_2_	171058–17–6	202.724	0.98	
Monoethanolamine	MEA	C_2_H_7_NO	141–43–5	61.08	0.995	Shanghai Macklin Biochemical Co., Ltd.
Ethanol	EtOH	C_2_H_6_O	64–17–5	46.068	0.995	Meryer (Shanghai) Biochemical Technology Co., Ltd.
3 Å molecular sieves	--	K*_n_* Na_12–*n*_[(AlO_2_)_12_(SiO_2_)_12_]·*x*H_2_O	308080–99–1			Shanghai Macklin Biochemical Co., Ltd.
Water	H_2_O	H_2_O	7732–18–5	0.0180	ultrapure (5.5 × 10^–6^ S/m)	--

The DESs were prepared by mixing the ILs of [EmimCl], [BmimCl], and [HmimCl] as HBAs and MEA as an HBD at a molar ratio of 1:4, followed by stirring under heating at 358 K for at least 2 h in the vacuum glovebox until homogeneous liquids were formed.^28^ To ensure accurate stoichiometry, all DESs were prepared gravimetrically using an analytical balance. For example, [EmimCl]­[MEA] (1:4) was prepared by mixing 30.0030 g of [EmimCl] with 49.9917 g of MEA, yielding a calculated molar ratio of MEA-to-IL in 3.9997 ± 0.0013. Similarly, the MEA-to-IL molar ratios for [BmimCl]­[MEA] (1:4) and [HmimCl]­[MEA] (1:4) were determined to be 4.0020 ± 0.0013 and 4.0011 ± 0.0014, respectively. While the actual molar ratios were controlled to a precision of 0.001, a nominal ratio of 1:4 was adopted for all DESs herein to ensure clarity. The resulting [EmimCl]­[MEA] (1:4) and [BmimCl]­[MEA] (1:4) are colorless and transparent, while [HmimCl]­[MEA] (1:4) appears light-yellow, transparent. In this work, the molar masses of DESs were calculated as follows. The results are summarized in Table 2

1 $$M_{\mathrm{DES}}=\frac{1}{5}M_{\mathrm{HBA}}+\frac{4}{5}M_{\mathrm{HBD}}$$

where *M*_HBA_ is the molar mass of [EmimCl], [BmimCl] or [HmimCl], and *M*_HBD_ is the molar mass of MEA.

**Table 2:** Molar Masses of the Studied DESs

	[EmimCl][MEA] (1:4)	[BmimCl][MEA] (1:4)	[HmimCl][MEA] (1:4)
Molar mass (g·mol^–1^)	79.19	83.80	89.41

After cooling to room temperature, the prepared DESs were stored in sealed vials containing 3Å molecular sieves to remove residual moisture. After storing for 2 weeks, the water content of each DES was determined by a Karl Fischer Moisture Meter (V100). Each sample was measured at least twice. The average water contents of these three prepared DESs are 280, 285, and 215 ppm, respectively.

### 2.2 Characterization of DESs

The successful formation of the DESs was confirmed through structural and thermal characterization. Fourier-transform infrared spectroscopy (FT-IR) and nuclear magnetic resonance (^1^H and ^13^C NMR) of the synthesized DESs (Figures S1–S5) revealed the formation of H-bonding interactions between the ILs and MEA. Thermal properties were examined by differential scanning calorimetry (DSC) to determine melting temperatures (*T*_m_). During the experiments, each sample (5–10 mg) was heated from 183 to 423 at 10 K·min^–1^ under a nitrogen atmosphere (40 mL·min^–1^). Considering the instrument accuracy of 0.1 K and the calibration residuals, the expanded uncertainty (*k* = 2) for the measured *T*_m_ was determined to be 0.2 K. For [EmimCl]­[MEA] (1:4) and [BmimCl]­[MEA] (1:4), the DSC thermograms were taken from our previous work and shown as Figure S6.^24^ From these thermograms, the melting points were determined to be 264.99 and 267.04 K, both of which are lower than the temperatures predicted by the ideal liquidus lines shown in Figure S7. The ideal liquidus lines of the (IL + MEA) mixtures have been calculated; the derivation equation and results are provided in the Supporting Information. According to the definition of DES, for a eutectic mixture composed of two or more components, the actual eutectic temperature should be lower than that of an ideal liquid mixture.^31^ Therefore, the thermal analysis supports classifying [EmimCl]­[MEA] (1:4) and [BmimCl]­[MEA] (1:4) as DESs. In contrast, [HmimCl] remains a supercooled liquid state under the DSC conditions, preventing direct determination of *T*_m_. Nevertheless, the spectroscopic features of [HmimCl]­[MEA] (1:4) are consistent with those of the other two systems, indicating the presence of analogous H-bonding interactions. Overall, the combination of the spectroscopic and thermal evidence supports the successful formation of [EmimCl]­[MEA] (1:4), [BmimCl]­[MEA] (1:4), and [HmimCl]­[MEA] (1:4) as DESs, consistent with previous reports.^24,32^

### 2.3 Preparation of the (DES + EtOH) Systems

The (DES + EtOH) mixtures were prepared gravimetrically inside a vacuum glovebox by weighing calculated amounts of DES and EtOH using an analytical balance (Sartorius SECURA225D-1CN) with an accuracy of 1 × 10^–4^ g. The prepared samples were sealed in glass vials and stored in a desiccator. Prior to measurements, the samples were stirred using a magnetic stirrer for over 2 h to ensure homogeneous mixing. The combined standard uncertainty in mass was *U*(*m*) = 5 × 10^–4^ g (0.95 level of confidence). Considering the final purity of the reagents (≥0.98), the combined standard uncertainty in mole fraction was approximately *u*(*x*) ≈ 0.004.

### 2.4 Measurements of Density (ρ), Viscosity (η), and Enthalpy of Mixing (Δ_mix_ *H*)

Densities (ρ) were measured with a vibrating U-tube density meter (Anton Paar DMA 5000M), with an accuracy of 7 × 10^–6^ g·cm^–3^ and repeatability of 1 × 10^–6^ g·cm^–3^. The instrument features temperature control with an uncertainty of 0.01 K. The instrument was calibrated using dry air and deionized water before the measurements, with the allowable density calibration error of 5 × 10^–5^ g·cm^–3^. Bubble-free operation was ensured using the built-in detection system. Each sample was measured at least twice, and average values were reported. The combined standard uncertainty in density was 4.7 × 10^–6^ g·cm^–3^, corresponding to an expanded uncertainty of 1 × 10^–5^ g·cm^–3^ (0.95 level of confidence). Since the sample purity affects the density measurement,^33^ the relative uncertainty in density was *U*_r_(ρ) ≈ 0.002.

Viscosities (η) were determined using a falling-ball viscometer (Anton Paar Lovis 2000 ME), with a specified accuracy of 0.5% and repeatability of 0.1%, employing a capillary tube with an internal diameter of 1.59 mm. The viscometer provides temperature control with an accuracy of 0.02 K and was calibrated with deionized water before use. Each measurement was performed at least twice, and the mean value was reported, yielding the relative uncertainty of viscosity to be *U*_r_(η) ≈ 0.017 (0.95 level of confidence).

Enthalpies of mixing (Δ_mix_ *H*) of DESs with cosolvents were measured using an isothermal titration calorimeter (TAM, TA Instruments, USA). Heat flow generated during mixing was integrated to obtain Δ_mix_ *H*. The instrument features air-circulated temperature control, ensuring thermal stability within ±0.02 K, and a heat-flow detection limit of 4 μW. During the experiment, a known amount of DES was placed in the ampule, and the cosolvent was loaded into a Hamilton syringe. The assembled unit was inserted into the experimental channel, and after 8 h baseline stabilization, the cosolvent was injected under continuous automated stirring to ensure complete mixing. The reference channel contained an equivalent premixed DES and cosolvent sample prepared 24 h in advance and stored at ambient temperature. After the experiment, the heat flow curves from each injection were integrated to obtain the heat exchange (*Q*), and Δ_mix_ *H* was calculated by dividing *Q* by the total moles of DES and cosolvent. Each composition was measured at least twice, and average values with associated uncertainties were reported.

## 3 Results and Discussion

### 3.1 Comparison of Measurement

To validate the accuracy of the density and viscosity measurements, the experimental data measured in this work were compared with literature values. In our previous work,^24^ the measured densities and viscosities of MEA and [BmimCl]­[MEA] (1:4) had confirmed their reliability, and thus, in the present study, EtOH was selected for further comparison. The density and viscosity of EtOH were measured at 1.01 × 10^5^ Pa over 288.15–323.15 K and compared with literature data. The temperature dependence of the EtOH density was fitted using eq 2

2 $$\rho_{\mathrm{fit}}=a_{\rho }+b_{\rho }T$$

where *T* represents temperature, and *a*_ρ_ and *b*_ρ_ are the fitting parameters. The resulting fitted parameters and average relative deviations (ARDs) are summarized in Table S1. The viscosity of EtOH was correlated with temperature using eq 3

3 $$\ln\left(\eta_{\mathrm{fit}}/\eta_{0}\right)=a_{\eta }+b_{\eta }/\left(c_{\eta }+T\right)\left(\eta_{0}=1\;\mathrm{mPa}\cdot s\right)$$

where *a*_η_, *b*_η_, and *c*_η_ are the fitting parameters. The resulting fitted parameters and ARDs are provided in Table S2. The relative deviations were calculated based on the fitted and literature values of density and viscosity using eq 4

4 $$100\delta =\frac{\left(Y_{\exp}-Y_{\mathrm{fit}}\right)}{Y_{\mathrm{fit}}},\left(Y=\rho ,\eta \right)$$

Figure 1 compares experimental densities with literature data, showing ARDs within 0.1%, consistent with the experimental uncertainty. Figure 2 compares the experimental and literature data on viscosity, with ARDs within 2%. Overall, the agreement confirms the reliability of the density and viscosity measured in this study.

**Figure 1:**
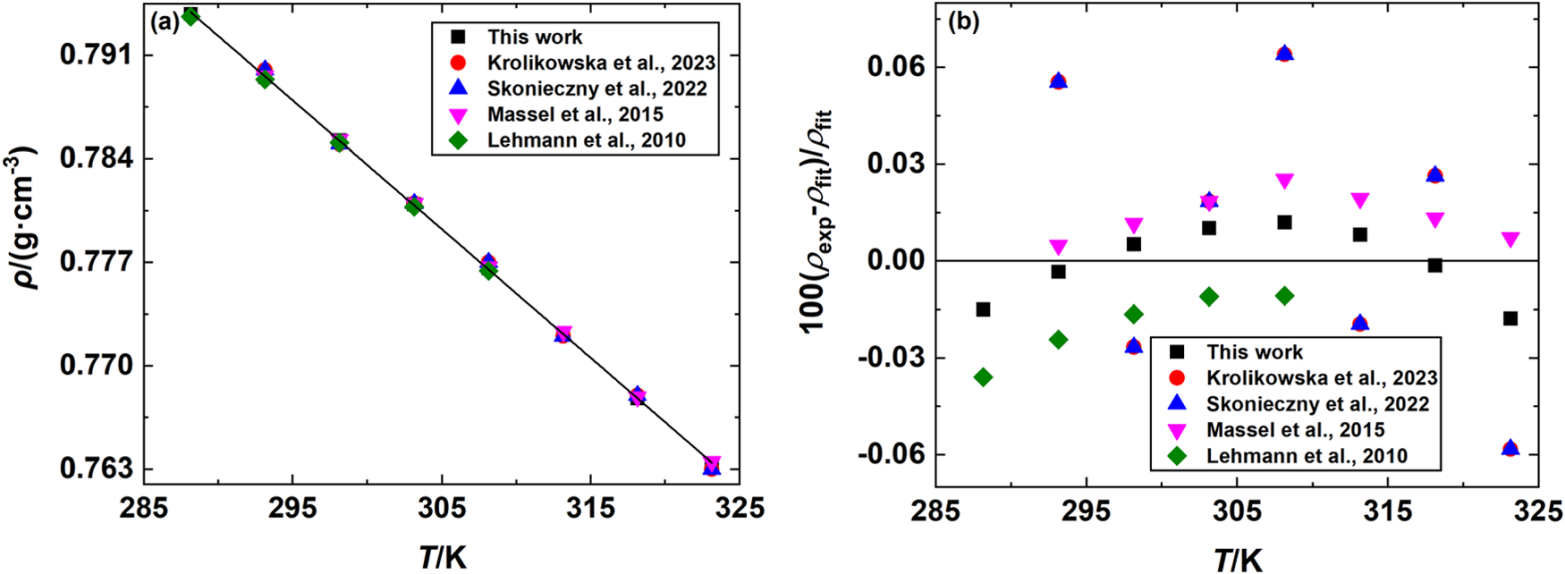
Comparison of experimental and literature density values from 288.15 to 323.15 K for EtOH^34–37^ with the corresponding deviations shown in (b). The solid black line in (a) represents the fitting results using eq 2, while that in (b) represents the zero line.

**Figure 2:**
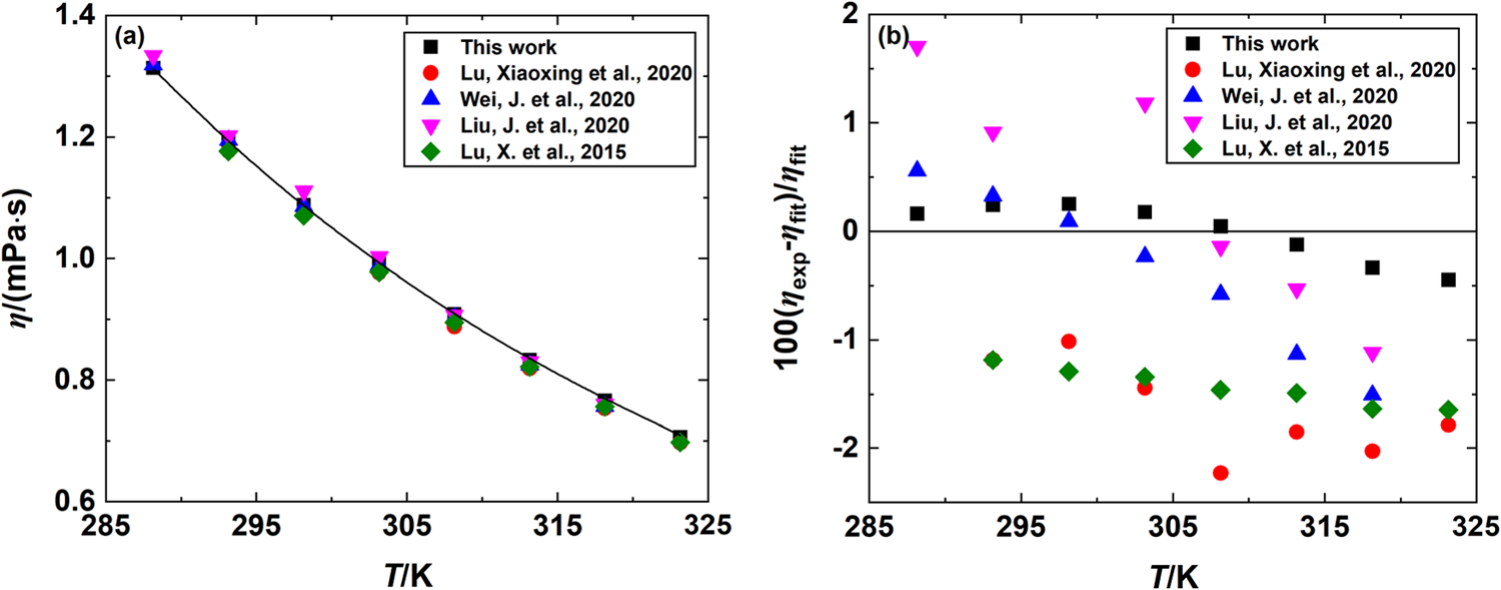
Comparison of experimental and literature values on viscosity from 288.15 to 323.15 K for EtOH^38–41^ (a) with the corresponding deviations shown in (b). The solid black line in (a) represents the fitting results using eq 3, while that in (b) represents the zero line.

To verify the accuracy and reliability of the Δ_mix_ *H* measurements, the enthalpy of mixing for the (EtOH + H_2_O) system at 298.15 K was determined. The results are summarized in Table S3 and presented in Figure 3. The experimental results showed good agreement with the reported data,^42,43^ thus demonstrating the reliability of the Δ_mix_ *H* measurement.

**Figure 3:**
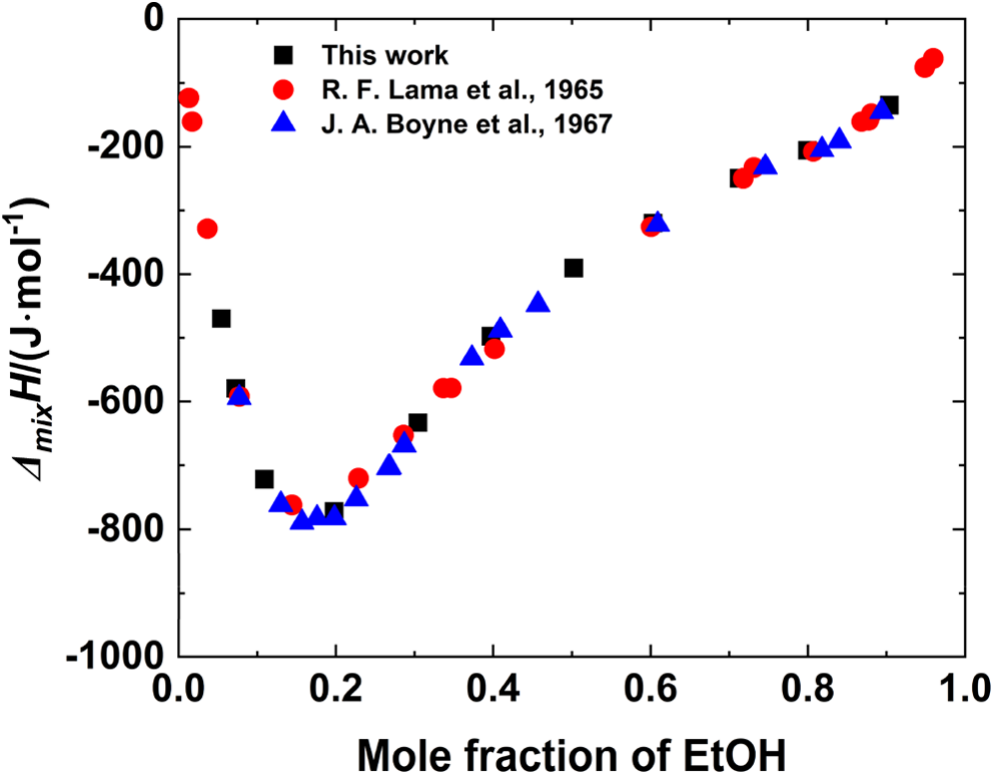
Comparison of experimental and literature values for the enthalpy of mixing of (EtOH + H_2_O) at 298.15 K.^42,43^

### 3.2 Density (ρ), Excess Molar Volume (*V* ^E^) and Partial Molar Volume (*V̅*_*i*_^E^)

The ρ of the three systems, i.e., ([EmimCl]­[MEA] (1:4) + EtOH), ([BmimCl]­[MEA] (1:4) + EtOH), and ([HmimCl]­[MEA] (1:4) + EtOH), were measured over the full compositional range at temperatures from 288.15 to 323.15 K in 5 K intervals, as summarized in Table 3.

**Table 3:** Densities (ρ) of ([EmimCl]­[MEA] (1:4) + EtOH), ([BmimCl]­[MEA] (1:4) + EtOH), and ([HmimCl]­[MEA] (1:4) + EtOH) at Different Mole Fractions (*x*_1_) and Temperatures (*T*) at 1.01 × 10^5^ Pa^a^

	*T* (K)							
*x*_1_	288.15	293.15	298.15	303.15	308.15	313.15	318.15	323.15
ρ (g·cm^–3^) of [EmimCl][MEA] (1:4) (1) + EtOH (2)								
0.000	0.79380	0.78954	0.78525	0.78094	0.77660	0.77221	0.76779	0.76331
0.100	0.83334	0.82923	0.82511	0.82097	0.81681	0.81262	0.80840	0.80414
0.205	0.86993	0.86592	0.86191	0.85788	0.85384	0.84978	0.84570	0.84160
0.302	0.90132	0.89740	0.89346	0.88952	0.88556	0.88160	0.87761	0.87362
0.401	0.93084	0.92697	0.92310	0.91922	0.91534	0.91145	0.90755	0.90363
0.500	0.95800	0.95417	0.95035	0.94653	0.94270	0.93888	0.93504	0.93120
0.600	0.98310	0.97931	0.97553	0.97175	0.96797	0.96419	0.96041	0.95663
0.704	1.00765	1.00389	1.00014	0.99640	0.99266	0.98893	0.98520	0.98147
0.800	1.02830	1.02458	1.02086	1.01715	1.01345	1.00975	1.00607	1.00238
0.896	1.04784	1.04414	1.04046	1.03678	1.03311	1.02945	1.02580	1.02216
ρ (g·cm^–3^) of [BmimCl][MEA] (1:4) (1) + EtOH (2)								
0.000	0.79380	0.78954	0.78525	0.78094	0.77660	0.77221	0.76779	0.76331
0.103	0.83371	0.82962	0.82551	0.82139	0.81724	0.81307	0.80887	0.80463
0.201	0.86721	0.86323	0.85923	0.85523	0.85120	0.84717	0.84311	0.83903
0.302	0.89812	0.89422	0.89031	0.88640	0.88248	0.87854	0.87459	0.87062
0.397	0.92457	0.92074	0.91690	0.91305	0.90920	0.90534	0.90148	0.89760
0.501	0.95083	0.94704	0.94326	0.93948	0.93570	0.93191	0.92813	0.92433
0.574	0.96761	0.96385	0.96011	0.95637	0.95263	0.94888	0.94514	0.94139
0.686	0.99149	0.98779	0.98409	0.98039	0.97670	0.97302	0.96934	0.96566
0.800	1.01335	1.00968	1.00602	1.00237	0.99873	0.99509	0.99146	0.98784
0.906	1.03194	1.02827	1.02462	1.02099	1.01740	1.01383	1.01028	1.00673
ρ (g·cm^–3^) of [HmimCl][MEA] (1:4) (1) + EtOH (2)								
0.000	0.79380	0.78954	0.78525	0.78094	0.77660	0.77221	0.76779	0.76331
0.097	0.83626	0.83213	0.82797	0.82380	0.81961	0.81538	0.81113	0.80684
0.199	0.87607	0.87202	0.86796	0.86389	0.85980	0.85570	0.85157	0.84741
0.301	0.91120	0.90722	0.90323	0.89923	0.89522	0.89119	0.88715	0.88309
0.399	0.94180	0.93787	0.93394	0.93000	0.92605	0.92209	0.91812	0.91414
0.497	0.96921	0.96531	0.96142	0.95753	0.95363	0.94973	0.94582	0.94190
0.601	0.99562	0.99176	0.98791	0.98406	0.98021	0.97635	0.97249	0.96863
0.698	1.01830	1.01448	1.01065	1.00683	1.00301	0.99920	0.99538	0.99157
0.801	1.04006	1.03626	1.03247	1.02868	1.02490	1.02112	1.01734	1.01357
0.894	1.05835	1.05458	1.05081	1.04705	1.04330	1.03955	1.03581	1.03207

a Standard uncertainties are *u*(*x*_1_) = 0.0015, *u*(*T*) = 0.01 K, *u*(*p*) = 3 kPa. The relative expanded uncertainty is *U*_r_(ρ) ≈ 0.002 (0.95 level of confidence).

The ρ is primarily governed by molecular packing and free volume.^44^ Because ρ exhibits similar trends with temperature and composition, only representative data are shown in Figure 4. As illustrated in Figure 4a, the ρ value of all the systems decreases linearly with increasing temperature. The variations of ρ at low *x*_1_ are more pronounced than those at high DES contents, indicating greater sensitivity to composition in the DES-lean (EtOH-rich) region due to more significant changes in microscopic structure and intermolecular interactions. Figure 4b presents ρ as a function of *x*_1_ at 288.15 and 323.15 K, revealing that ρ increases with increasing the DES content. For pure DESs and the (DES + EtOH) systems, at a given temperature, ρ follows the order: [HmimCl]­[MEA] (1:4) > [EmimCl]­[MEA] (1:4) > [BmimCl]­[MEA] (1:4). This trend contrasts with that of the corresponding pure imidazolium-based ILs,^45^ where ρ decreases with increasing cation alkyl chain length due to enlarged cation volume and reduced packing efficiency.^46^ This behavior likely arises from a balance between alkyl-chain-induced volume expansion, which hinders compact packing, and enhanced intermolecular interactions (e.g., between [HmimCl] and MEA), which promote denser packing.

**Figure 4:**
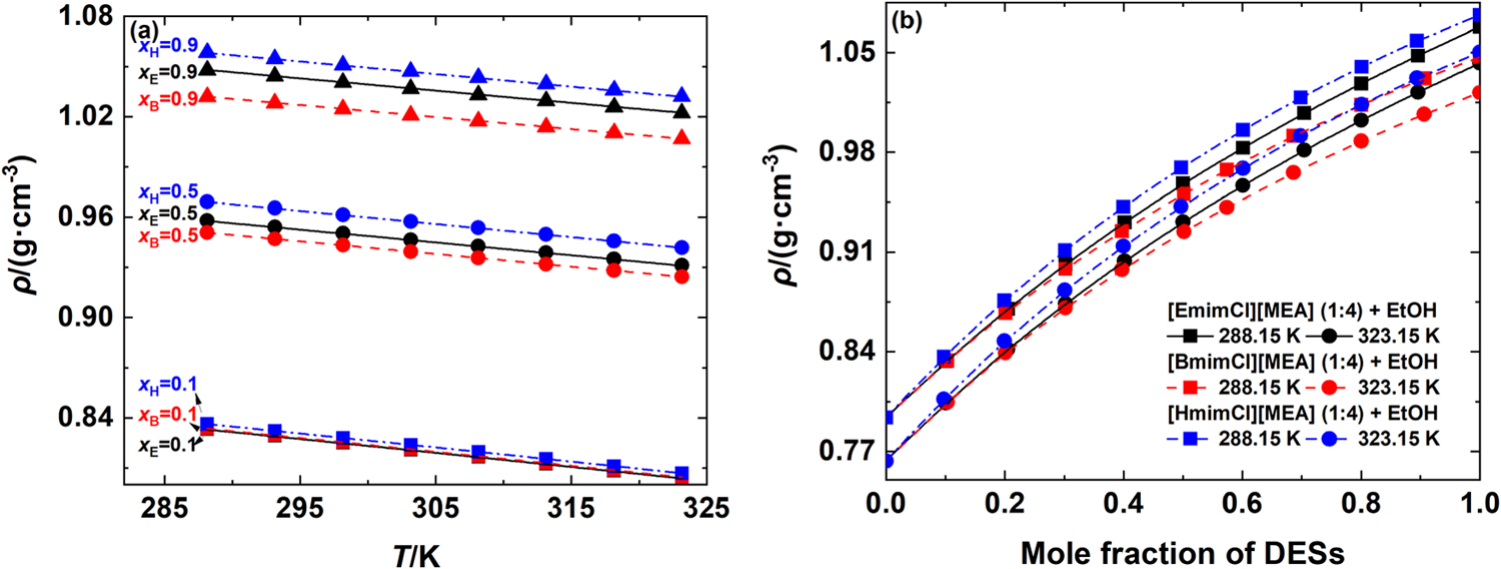
(a) Density (ρ) of the studied (DES + EtOH) as a function of temperature (*T*) at the DES mole fractions of 0.1, 0.5, and 0.9. The variables *x*_E,_ *x*_B_, and *x*_H_ represent the mole fractions of [EmimCl]­[MEA] (1:4), [BmimCl]­[MEA] (1:4), and [HmimCl]­[MEA] (1:4), respectively. (b) ρ of the studied (DES + EtOH) as a function of the DES mole fraction at 288.15 and 323.15 K. Curves are provided as visual guides.

To assess the nonideality of DES–solvent interactions, the excess molar volumes (*V* ^E^) of the (DES + EtOH) systems were calculated using eq 5

5 $$V^{E}=\frac{x_{1}M_{1}+x_{2}M_{2}}{\rho }-\frac{x_{1}M_{1}}{\rho_{1}^{*}}-\frac{x_{2}M_{2}}{\rho_{2}^{*}}$$

where *x*_*i*_ and *M*_*i*_ are the mole fraction and molecular weight of component *i*; ρ, ρ_1_*, and ρ_2_* are the densities of the mixture, pure DES, and pure EtOH, respectively. The calculated *V* ^E^ values are listed in Table S4. The composition dependence of *V* ^E^ was correlated using the RK polynomial equation, with fitted parameters and corresponding ARDs summarized in Table S5.

6 $$V^{E}=x_{1}x_{2}\sum_{j=0}^{k}A_{j}{\left(x_{1}-x_{2}\right)}^{j}$$

where *A*_*j*_ is the RK coefficients and *k* denotes the polynomial order, fixed at 4 in this study. Despite these empirical parameters lacking strict physical meaning, they can provide qualitative insight into nonideality and intermolecular interactions. For example, *A*_1_ represents the magnitude and direction of the deviations, while higher-order parameters (*A*_2_ – *A*_5_) describe asymmetry and composition dependence of the systems.

Figure 5 shows the *V* ^E^ value of the (DES + EtOH) systems as a function of *x*_1_. For all systems, *V* ^E^ remains negative over the entire compositional range, indicating that volume contraction. This behavior is mainly attributed to ion–dipole interactions between EtOH and the DES components, as well as strong H-bonding interactions between the −OH group of EtOH and the −NH_2_ and −OH groups of MEA (e.g., −H_2_N···HO– and −HO···HO−), which promote more efficient molecular packing within the studied systems. The composition dependence of *V* ^E^ for the studied systems is similar to that observed for the ([HmimCl] + EtOH) and (MEA + EtOH) systems.^47,48^ The largest absolute *V* ^E^ values are observed in the EtOH-rich region (*x*_1_ ≈ 0.4), consistent with the more pronounced density changes in this compositional range.

**Figure 5:**
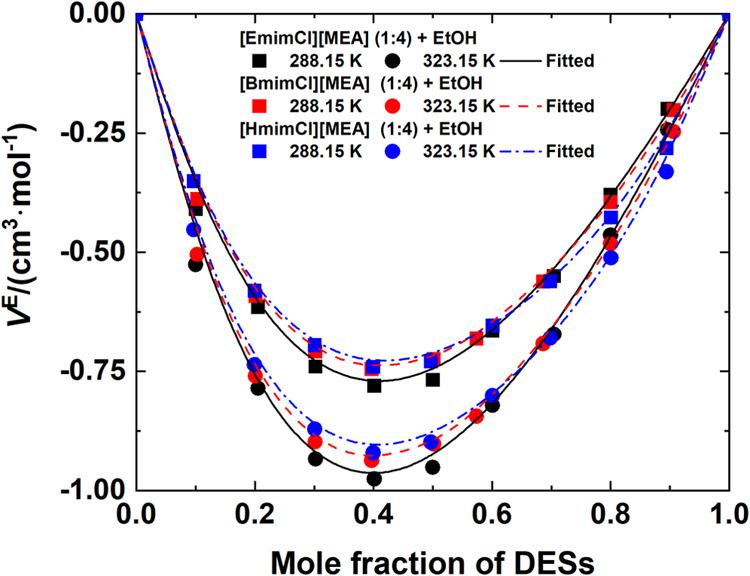
Excess volumes (*V* ^E^) of the studied (DES + EtOH) as a function of the DES mole fraction at 288.15 and 323.15 K. Symbols: experimental data; Curves: results of the RK polynomial expression.

As *x*_1_ increases, *V* ^E^ initially decreases and then increases, exhibiting a characteristic “U-shaped” trend. The minimum *V* ^E^ occurs at *x*_1_ ≈ 0.4, corresponding to the greater density change in the EtOH-rich region. Moreover, as the temperature increases, the *V* ^E^ of the (DES + EtOH) systems becomes increasingly negative, indicating that molecular packing efficiency plays a more dominant role than H-bonding interactions. The magnitude of |*V* ^E^| follows the order of ([EmimCl]­[MEA] (1:4) + EtOH) > ([BmimCl]­[MEA] (1:4) + EtOH) > ([HmimCl]­[MEA] (1:4) + EtOH), indicating that the extent of volume contraction decreases with increasing alkyl chain length of the IL cation. DESs with shorter alkyl chains exhibit lower steric hindrance, which facilitates stronger ion–dipole interactions and H-bonding with EtOH.

Partial molar volume (*V̅_i_*) reflects the actual volumetric contribution of the component in the mixtures, while excess partial molar volume (*V̅*_*i*_^E^) represents the deviation of this contribution from that in the pure-component reference state. Both *V̅_i_* and *V̅*_*i*_^E^ are important thermodynamic quantities for providing insight into the intermolecular interactions between DES and EtOH and the nonideal behavior in the studied (DES + EtOH) systems.^49^ In this work, to further study the volumetric behavior of the systems, the *V̅_i_* and *V̅*_*i*_^E^ of the individual components were calculated using eqs 7 and 8, respectively.

7 $${\mathrm{V̅}}_{i}=V_{m,i}+V^{E}+\left(1-x_{i}\right){\left(\partial V^{E}/\partial x_{i}\right)}_{P,T}$$

8 $${\mathrm{V̅}}_{i}^{E}={\mathrm{V̅}}_{i}-V_{m,i}$$

where *V*_*m*,*i*_ is the molar volume of pure component *i*, and (∂*V* ^E^/∂*x*_*i*_)_*P*,*T*_ was obtained from the parameters of the fourth-order polynomial density correlation. Since the *V̅_i_* and *V̅*_*i*_^E^ differ only by the molar volume of the pure component, only the *V̅*_*i*_^E^ of the studied (DES + EtOH) systems were reported in this study, as summarized in Tables S6–S8.

Figure 6 shows the variations of the *V̅*_*i*_^E^ as a function of *x*_1_ at 288.15 K. The *V̅*_*i*_^E^ of both DES and EtOH are negative over the full compositional range. This result is consistent with the negative *V* ^E^ discussed above, suggesting volume contraction upon mixing. The negative values of *V̅*_*i*_^E^ suggest strong DES–EtOH interactions and efficient molecular packing in the studied systems. As *x*_*i*_ increases, the values of *V̅*_*i*_^E^ increase, indicating strong intermolecular interactions between the constituents of DES and EtOH make their volume in EtOH less than that of the pure DES. In addition, the alkyl chain length of ILs has only slight influence on *V̅*_1_^E^ and *V̅*_2_^E^.

**Figure 6:**
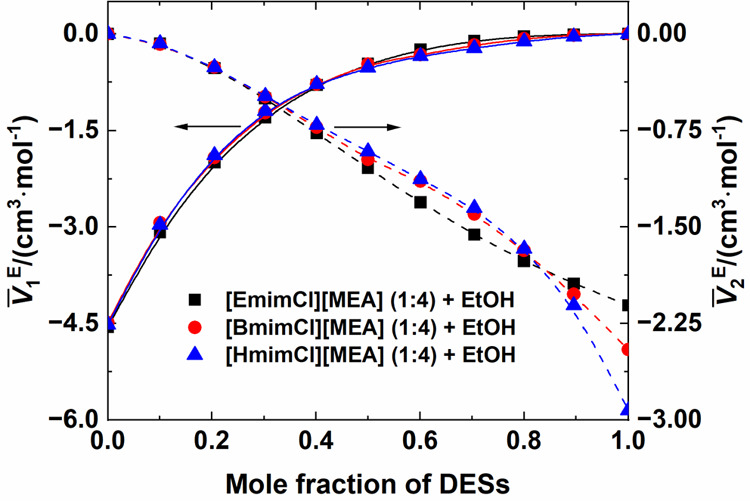
Excess partial molar volumes (*V̅*_*i*_^E^) of the studied (DES + EtOH) as a function of the DES mole fraction at 288.15 K. *V̅*_1_^E^ and *V̅*_2_^E^ represent the excess partial molar volumes of DES and EtOH in the (DES + EtOH) systems, respectively. Curves are provided as visual guides.

### 3.3 Viscosity (η), Viscosity Deviation (Δη) and Excess Molar Gibbs Energy of Activation (G*^,E^)

The η values of the three studied (DES + EtOH) systems were measured over the full compositional range at temperatures ranging from 288.15 to 323.15 K in 5 K intervals. The results are presented in Table 4.

**Table 4:** Viscosities (η) of ([EmimCl]­[MEA] (1:4) + EtOH), ([BmimCl]­[MEA] (1:4) + EtOH), and ([HmimCl]­[MEA] (1:4) + EtOH) at Different Mole Fractions (*x*_1_) and Temperatures (*T*) at 1.01 × 10^5^ Pa^a^

	*T* (K)							
*x*_1_	288.15	293.15	298.15	303.15	308.15	313.15	318.15	323.15
η (mPa·s) of [EmimCl][MEA] (1:4) (1) + EtOH (2)								
0.000	1.31	1.19	1.09	0.99	0.91	0.83	0.77	0.71
0.100	1.98	1.79	1.61	1.46	1.33	1.21	1.11	1.01
0.205	2.86	2.56	2.30	2.07	1.86	1.69	1.53	1.40
0.302	4.04	3.55	3.14	2.79	2.49	2.23	2.01	1.82
0.401	5.76	4.99	4.32	3.79	3.36	2.99	2.67	2.39
0.500	7.99	6.82	5.86	5.07	4.43	3.89	3.45	3.06
0.600	10.77	9.03	7.64	6.52	5.62	4.89	4.29	3.80
0.704	14.83	12.15	10.10	8.50	7.24	6.22	5.39	4.72
0.800	20.13	16.24	13.30	11.01	9.25	7.86	6.74	5.84
0.896	27.30	21.56	17.34	14.16	11.73	9.84	8.36	7.17
η (mPa·s) of [BmimCl][MEA] (1:4) (1) + EtOH (2)								
0.000	1.31	1.19	1.09	0.99	0.91	0.83	0.77	0.71
0.103	2.07	1.87	1.69	1.53	1.39	1.27	1.16	1.06
0.201	2.99	2.67	2.39	2.15	1.94	1.75	1.59	1.45
0.302	4.38	3.83	3.38	2.99	2.66	2.38	2.14	1.93
0.397	6.21	5.36	4.66	4.08	3.59	3.19	2.84	2.54
0.501	9.12	7.72	6.59	5.67	4.93	4.31	3.80	3.37
0.574	11.85	9.89	8.34	7.09	6.10	5.28	4.61	4.06
0.686	17.70	14.48	11.95	9.99	8.43	7.19	6.20	5.38
0.800	25.25	20.15	16.29	13.39	11.16	9.38	7.99	6.87
0.906	34.70	27.60	21.95	17.94	14.81	12.30	10.34	8.78
η (mPa·s) of [HmimCl][MEA] (1:4) (1) + EtOH (2)								
0.000	1.31	1.19	1.09	0.99	0.91	0.83	0.77	0.71
0.097	2.00	1.81	1.64	1.48	1.35	1.23	1.12	1.03
0.199	2.98	2.66	2.37	2.13	1.91	1.73	1.57	1.43
0.301	4.44	3.90	3.43	3.04	2.71	2.42	2.17	1.96
0.399	6.57	5.66	4.90	4.27	3.75	3.31	2.95	2.63
0.497	9.15	7.68	6.51	5.57	4.82	4.24	3.72	3.29
0.601	13.51	11.01	9.15	7.70	6.56	5.64	4.89	4.31
0.698	20.06	16.34	13.40	11.12	9.35	7.95	6.82	5.90
0.801	28.43	22.60	18.09	14.76	12.19	10.21	8.67	7.42
0.894	39.14	30.37	24.05	19.42	15.85	13.10	11.00	9.33

a Standard uncertainties are *u*(*x*_1_) = 0.0015, *u*(*T*) = 0.01 K, u­(*p*) = 3 kPa. The relative expanded uncertainty is *U*_r_(η) ≈ 0.017 (0.95 level of confidence).

The η is dominated by intermolecular interactions, particularly the H-bonding interactions between HBA and HBD components.^50^ Figure 7a illustrates the temperature dependence of the η for the (DES + EtOH) systems at *x*_1_ of 0.1, 0.5, and 0.9. For all systems, η decreases exponentially with increasing temperature, consistent with Arrhenius-type behavior. In contrast to ρ, η exhibits greater sensitivity to *x*_1_ in the DES-rich region, indicating a stronger compositional influence on viscous behavior. Figure 7b displays the η as a function of *x*_1_ at 288.15 and 323.15 K. The η increases exponentially with increasing DES content. At a given temperature, the η values follow the order: ([HmimCl]­[MEA] (1:4) + EtOH) > ([BmimCl]­[MEA] (1:4) + EtOH) > ([EmimCl]­[MEA] (1:4) + EtOH), indicating that η increases with alkyl chain length of the imidazolium cation. This trend is consistent with that reported for pure ILs by Fendt et al.,^51^ where η increases with increasing cation alkyl chain length due to enhanced van der Waals interactions.^52^ This increase may be attributed to the strengthening of intermolecular interactions (e.g., van der Waals forces and H-bonding), which raise resistance to flow.

**Figure 7:**
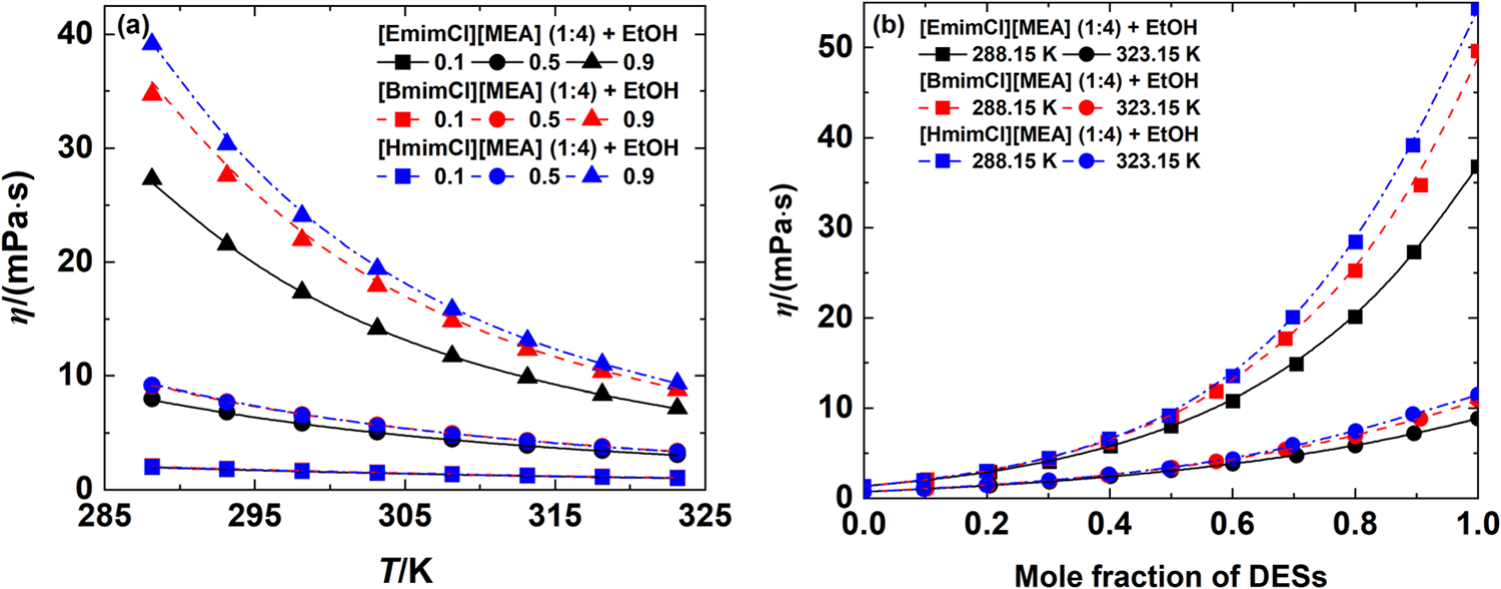
(a) Viscosity (η) of the studied (DES + EtOH) as a function of temperature (*T*) at the DES mole fractions of 0.1, 0.5, and 0.9. (b) η of the studied (DES + EtOH) as a function of the DES mole fraction at 288.15 and 323.15 K. Curves are provided as visual guides.

To further elucidate intermolecular interactions influencing the viscosities of the (DES + EtOH) systems, viscosity deviations (Δη) were calculated using eq 9. The resulting Δη values are listed in Table S9.

9 $$\Delta \eta =\eta -\left(x_{1}\eta_{1}+x_{2}\eta_{2}\right)$$

where η_1_ and η_2_ are the viscosities of the pure DES and EtOH, respectively; *x*_1_ and *x*_2_ are the mole fractions of DES and EtOH, respectively. The Δη for the (DES + EtOH) system was also correlated using the RK equation, as shown in eq 6. The parameters of the RK equation for the Δη correlation and the corresponding ARDs are presented in Table S10.

Figure 8 shows the Δη as a function of *x*_1_ for the (DES + EtOH) systems. Δη remains negative over the entire compositional range and exhibits a characteristic “U-shaped” profile. Given the strong H-bonding ability of EtOH, the negative deviations are attributed to the disruption of the internal H-bond network of the DES upon the addition of EtOH, which weakens the associated structure of the DES and increases molecular mobility, resulting in η lower than estimated by ideal mixing (*x*_1_η_1_ + *x*_2_η_2_). The minimum Δη occurs at approximately *x*_1_ ≈ 0.6, consistent with trends reported for the choline-based DES aqueous solutions.^53^ Based on molecular simulation studies for the choline-based DES aqueous systems,^19^ it can be inferred that the DESs are able to preserve their original H-bond network and microscopic structure to a certain extent until the EtOH content reaches approximately 0.4.

**Figure 8:**
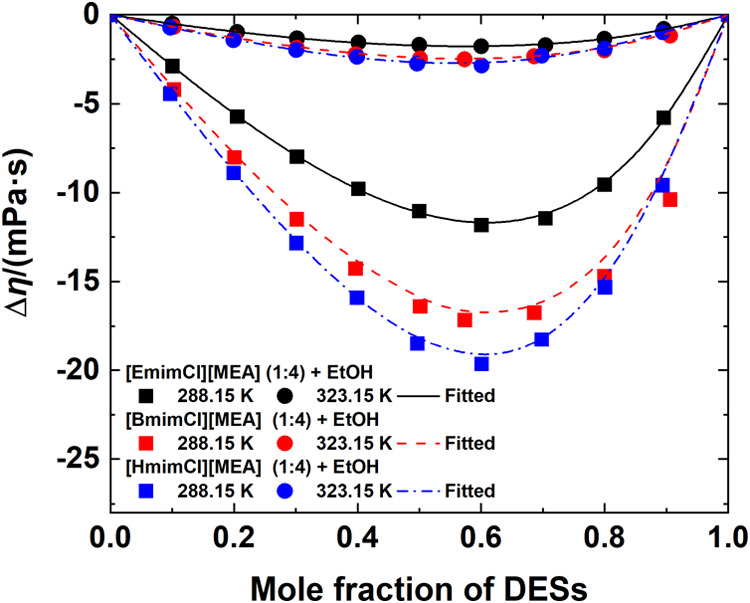
Viscosity deviations (Δη) of the studied (DES + EtOH) as a function of the DES mole fraction at 288.15 and 323.15 K. Symbols: experimental data; curves: results of the RK polynomial fitting.

As the temperature increases, the |Δη| values for the (DES + EtOH) systems decrease. The enhanced molecular motion and kinetic energy lead to the weakened intermolecular interactions, particularly H-bonding, thereby reducing the nonideality of the (DES + EtOH) systems. An increase in the |Δη| value is observed for the (DESs + EtOH) systems as the alkyl chain length of the ILs increases, suggesting that EtOH can more readily disrupt the original H-bond network in the DESs with longer alkyl chains. As a result, the flow resistance is reduced and molecular mobility increases, resulting in a greater negative Δη in the systems with longer alkyl chains.

Based on the Eyring’s absolute rate theory, the excess molar Gibbs energy of activation (G*^,E^),^54^ was calculated from the viscosity and molar volume (*V*) data using eq 10 to further evaluate the nonideal viscous flow behavior of the (DES + EtOH) mixtures. The *V* was calculated by dividing the molar mass (*M*) by the ρ.

10 $$G^{*,E}=\mathrm{RT}\left[\ln\left(\eta V\right)-x_{1}\ln\left(\eta_{1}V_{1}\right)-x_{2}\ln\left(\eta_{2}V_{2}\right)\right]$$

where *R* denotes the universal gas constant, *T* represents the temperature in Kelvin, and η_1_, η_2_, and η correspond to the viscosities of the DES, EtOH, and (DES + EtOH) systems, respectively. *V*_1_, *V*_2_, and *V* represent the molar volumes of the DES, EtOH, and (DES + EtOH) systems, respectively. The calculated G*^,E^ values are listed in Table S11, and their composition dependences at 288.15 and 323.15 K are shown in Figure 9. It is evident that the G*^,E^ values are positive over the entire compositional range, indicating that specific H-bonding interactions lead to the formation of complexes between the component molecules.^55^ The G*^,E^ values at 323.15 K are higher than those at 288.15 K, suggesting that the nonideal behavior becomes more pronounced at higher temperature. In addition, the alkyl chain length of the ILs has a limited influence on G*^,E^, and the magnitude approximately follows the order ([EmimCl]­[MEA] (1:4) + EtOH) ≈ ([BmimCl]­[MEA] (1:4) + EtOH) > ([HmimCl]­[MEA] (1:4) + EtOH). This indicates that the ([EmimCl]­[MEA] (1:4) + EtOH) and ([BmimCl]­[MEA] (1:4) + EtOH) systems exhibit more pronounced nonideal behavior, which is attributed to their shorter alkyl chain lengths.

**Figure 9:**
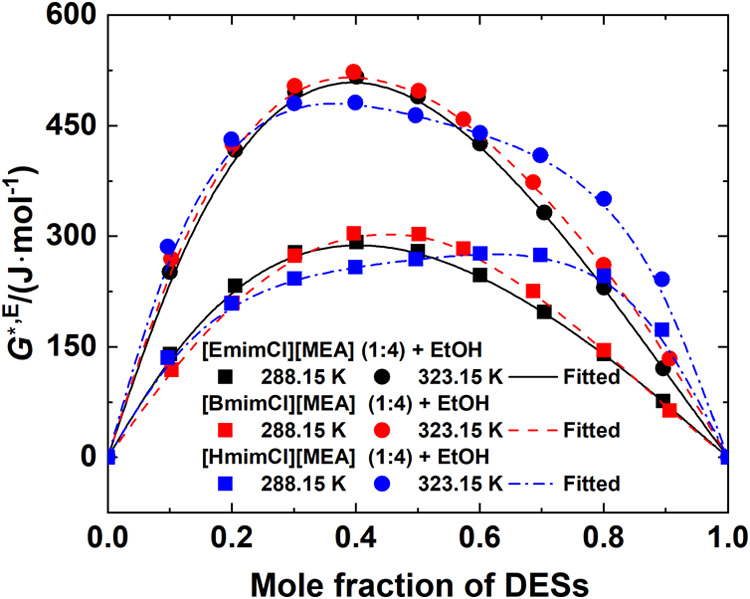
Excess molar Gibbs energy of activation (G*^,E^) of the studied (DES + EtOH) as a function of the DES mole fraction at 288.15 and 323.15 K. Curves are provided as visual guides.

### 3.4 Enthalpy of Mixing (Δ_mix_ *H*) and Thermodynamic Modeling

#### 3.4.1 Experimental Data

The Δ_mix_ *H* values of the (DES + EtOH) systems were measured over the full compositional range at 298.15 and 308.15 K. The measured values along with their associated uncertainties are listed in Table S12, and the composition dependence of Δ_mix_ *H* at both temperatures is shown in Figure 10. The Δ_mix_ *H* values were correlated using the RK equation in eq 6, with the fitted parameters and coefficient of determination (*R* ^2^) provided in Table S13. All systems exhibit a characteristic “S-shaped” dependence of Δ_mix_ *H* on composition, reflecting changes in intermolecular interactions during the mixing.

**Figure 10:**
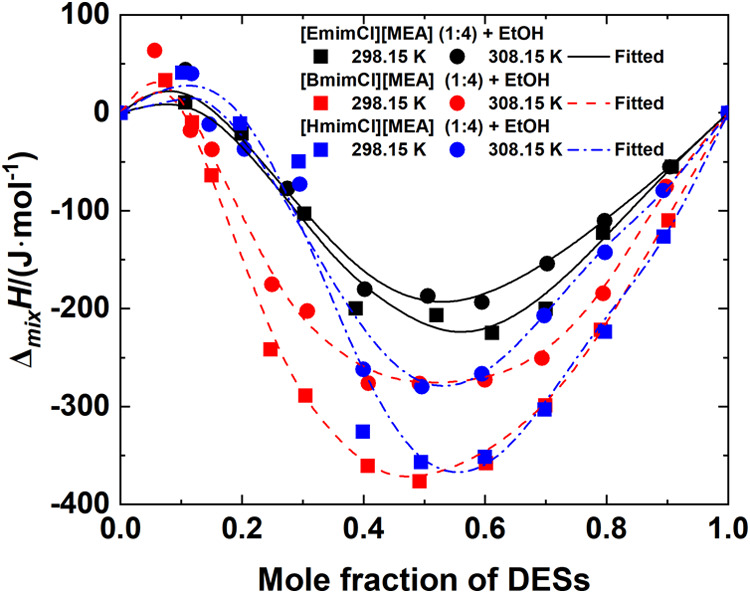
Enthalpies of mixing (Δ_mix_ *H*) of the studied (DES + EtOH) as a function of the DES mole fraction at 298.15 and 308.15 K. Symbols: experimental data; curves: results of the RK polynomial fitting.

At low *x*_1_ (*x*_1_ < 0.15), Δ_mix_ *H* is positive for all systems, indicating that interactions within the pure components (DES–DES and EtOH–EtOH) are stronger than those between DES and EtOH molecules. As *x*_1_ exceeds 0.15, Δ_mix_ *H* becomes negative, suggesting the formation of stronger intermolecular interactions, such as H-bonding, between the IL or MEA in DESs and EtOH molecules. These interactions likely disrupt the original H-bond network of the DES and promote the formation of new H-bonds.

The Δ_mix_ *H* curves at 308.15 K generally lie above those at 298.15 K, indicating that the increased temperature weakens the exothermic nature of the mixing process. This behavior can be attributed to the weakening of H-bonding interactions between DESs and EtOH at higher temperatures, leading to a decrease in the absolute value of Δ_mix_ *H*.

In contrast to the trends observed for ρ and η, the absolute values of Δ_mix_ *H* are largest for the ([BmimCl]­[MEA] (1:4) + EtOH) and smallest for the ([EmimCl]­[MEA] (1:4) + EtOH) systems. These findings highlight the complexity of alkyl-chain-length effects on the H-bond network, microstructural organization, and intermolecular interactions within the DESs upon the addition of EtOH, suggesting that further investigations, such as molecular dynamics simulations, would be useful to clarify these effects.

## 4 Further Comparison

Our previous work systematically measured ρ, η, and Δ_mix_ *H* for the systems of the three amine-based DESs ([EmimCl]­[MEA] (1:4), [BmimCl]­[MEA] (1:4), and [HmimCl]­[MEA] (1:4)) with H_2_O.^24^ In addition, Li et al.^56^ investigated the ρ, η, and excess properties of ([ChCl]­[MEA] + H_2_O) systems with ChCl:MEA molar ratios of 1:5, 1:6, 1:8, and 1:10. Since these systems exhibited similar trends in physical properties, the ([ChCl]­[MEA] (1:5) + H_2_O) system was selected as a representative system for comparison. To further clarify the effects of cosolvents and HBAs on the thermodynamic behavior of amine-based DESs, the ρ, η, *V* ^E^, and Δη values of the ([C*_n_* mimCl]­[MEA] (1:4) + H_2_O), ([C*_n_* mimCl]­[MEA] (1:4) + EtOH), and ([ChCl]­[MEA] (1:5) + H_2_O) systems were compared. In addition, the Δ_mix_ *H* values of the ([C*_n_* mimCl]­[MEA] (1:4) + H_2_O) and ([C*_n_* mimCl]­[MEA] (1:4) + EtOH) systems were also analyzed comparatively.

Figure 11a**–**b shows the variations in ρ and *V* ^E^ over the full compositional range as a function of the *x*_1_ of the (DES + EtOH) and (DES + H_2_O) systems, respectively. Overall, the ρ values of the (DES + H_2_O) systems are higher than those of the (DES + EtOH) systems, primarily due to H_2_O having a greater ρ than EtOH. It is noteworthy that the ρ value of the (DES + H_2_O) systems shows a nonmonotonic variation. This behavior may be attributed to the small molecular size and high polarity of H_2_O. At low H_2_O contents, the H_2_O molecules may penetrate into the original H-bond network of the DES, leading to more efficient molecular packing and thus a slight increase in ρ. However, as the H_2_O content increases further, the excess H_2_O may progressively disrupt the native H-bond network of the DES, weakening intermolecular associations and resulting in volumetric expansion. In contrast, the EtOH molecules are larger and contain a hydrophobic alkyl group, which may limit their ability to integrate into the DES H-bond network in the same manner as H_2_O. Rather than promoting structural compaction at low concentrations, EtOH appears to induce a more gradual modification of the DES structure, resulting in a more monotonic and nearly linear increase in ρ with increasing *x*_1_.

**Figure 11:**
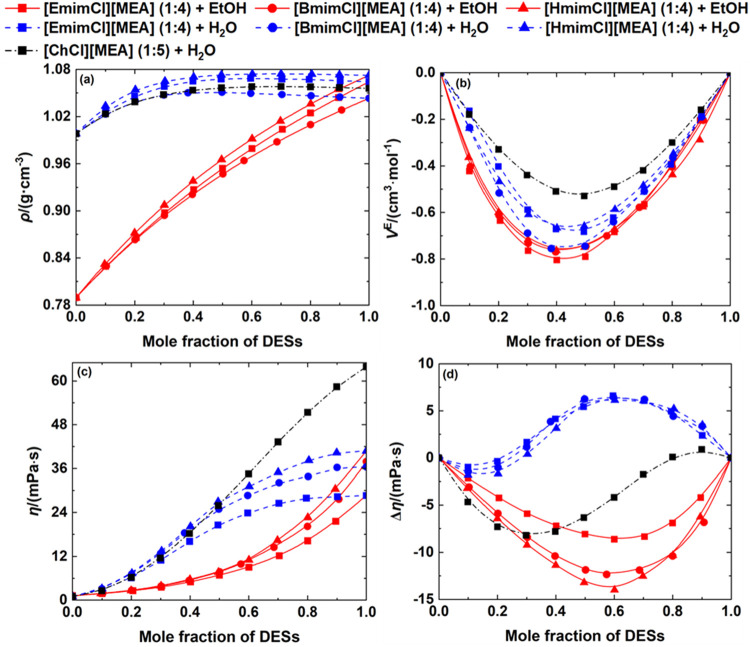
(a) Densities (ρ), (b) excess volumes (*V* ^E^), (c) viscosities (η), and viscosity deviations (Δη) of (DES + EtOH) and (DES + H_2_O) as a function of the DES mole fraction at 293.15 K. Symbols are experimental or literature data; curves are provided as visual guides.

Further analysis of *V* ^E^ provides insight into the origin of nonideality. The distinct temperature dependence of *V* ^E^ reveals that the nonideality in the (DES + H_2_O) systems is mainly governed by H-bond, while the (DES + EtOH) systems are more dominated by molecular packing effects. For the [EmimCl]­[MEA] (1:4) and [HmimCl]­[MEA] (1:4)-based systems, |*V* ^E^| is consistently larger when EtOH is served as the cosolvent compared to H_2_O, indicating stronger deviations from ideality in the (DES + EtOH) systems. Conversely, for the [BmimCl] [MEA] (1:4)-based system, the opposite trend is observed, with (DES + H_2_O) exhibiting a larger |*V* ^E^|. This contrasting behavior suggests that the interplay between the cation alkyl chain length and solvent characteristics jointly determines the extent and nature of nonideality in these mixtures. Compared with the ([C*_n_* mimCl]­[MEA] (1:4) + H_2_O) systems, the smaller |*V* ^E^| of the ([ChCl]­[MEA] (1:5) + H_2_O) system indicate a weaker volume contraction effect, which may be attributed to the more compact structure of the ChCl-based DES resulting from the smaller size of the [Ch]^+^ cation, which limits further volume reduction upon the addition of H_2_O. In addition, the shift of the minimum toward higher *x*_1_ indicates a relatively limited capacity of the H-bond network of the ChCl-based DES to accommodate H_2_O molecules.

Figure 11c**–**d shows the variations in η and Δη over the full compositional range as a function of *x*_1_ of the (DES + EtOH) and (DES + H_2_O) systems, respectively. The η values of the (DES + H_2_O) systems are generally higher than those of the corresponding (DES + EtOH) systems. In the DES-rich region, the η values of the (DES + EtOH) systems exhibit greater sensitivity to changes in the molar fraction of DES. This behavior may be related to the distinct roles of the cosolvents within the DES microstructure. As suggested by the analysis of ρ, the H_2_O molecules may partially integrate into the H-bond network of the DES and form relatively stable local structures, thereby limiting the reduction in η. In contrast, due to the larger molecular size and hydrophobic alkyl group of EtOH, its capability to integrate into the DES H-bond framework is lower. As a result, the structural connectivity of the DES network may be more readily reduced with increasing the EtOH content, which is reflected in a more pronounced decrease in η. Compared with the ([C*_n_* mimCl]­[MEA] (1:5) + H_2_O) systems, ([ChCl]­[MEA] (1:5) + H_2_O) system exhibits greater sensitivity to composition-dependent in the DES-rich region. This behavior arises from the structural characteristics of the HBA cations. Compared with the imidazolium cation, the smaller size and lower steric hindrance of [Ch]^+^ cation favor stronger intermolecular interactions (e.g., H-bonding) with MEA, resulting in a higher η value for the pure ChCl-based DES. Consequently, the addition of a small amount of H_2_O causes a more pronounced disruption of the original DES structure, leading to a sharper decrease in η in the DES-rich region.

In Figure 11d, Δη is examined to further quantify the nonideal mixing behavior. Notably, the (DES + H_2_O) mixtures display an “S-shaped” dependence of Δη on composition, suggesting a composition-dependent restructuring of the H-bond network rather than a simple dilution effect. Specifically, at low to intermediate DES fractions, the incorporation of a limited amount of H_2_O may promote the reorganization (and partial reinforcement) of local H-bond motifs, which can partly compensate for the viscosity reduction expected from bond cleavage. At higher H_2_O contents, however, the excess H_2_O is more likely to disrupt the DES-associated network connectivity, leading to a different sign/magnitude response and resulting in the overall “S-shaped” profile. By contrast, the (DES + EtOH) systems exhibit a consistently negative Δη with a broadly “U-shaped” dependence, implying that the mixing-induced structural modifications remain qualitatively similar throughout the compositional range. Accordingly, Δη remains negative across all compositions, reaching its maximum magnitude at intermediate mole fractions. Compared with the ([C*_n_* mimCl]­[MEA] (1:5) + H_2_O) systems, the transition from negative to positive deviation occurs at higher *x*_1_ in the ([ChCl]­[MEA] (1:5) + H_2_O) system, and |Δη| values are smaller at low H_2_O contents. This behavior suggests that the ChCl-based DES possesses a more compact H-bond network with a relatively limited capacity to accommodate H_2_O molecules. As the H_2_O content increases, the disruptive effect of excess H_2_O on the original H-bond network of the DES becomes more pronounced.

Figure 12 presents the variation of Δ_mix_ *H* as a function of *x*_1_ at 298.15 K for the (DES + EtOH) and (DES + H_2_O) systems. For the (DES + H_2_O) systems, Δ_mix_ *H* is consistently negative over the entire compositional range, and the magnitude of |Δ_mix_ *H*| is significantly larger than that of the corresponding (DES + EtOH) systems. This indicates stronger attractive interactions in the presence of H_2_O. The strong polarity and high dielectric constant of H_2_O induce extensive H-bond network reorganization, resulting in pronounced exothermic effects, marked volume contraction, and significant viscosity transitions. These factors collectively demonstrate that H_2_O drastically restructures the H-bond network of the DES compared to EtOH.

**Figure 12:**
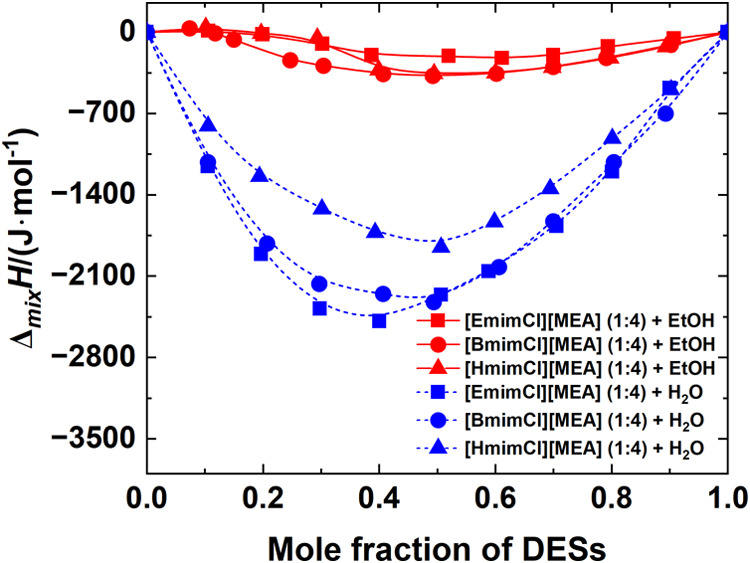
Enthalpies of mixing (Δ_mix_ *H*) of (DES + EtOH) and (DES + H_2_O) as a function of the DES mole fraction at 298.15 K. Curves are provided as visual guides.

## 5 Conclusion

The ρ and η of their mixtures of the three amine-based DESs ([EmimCl]­[MEA] (1:4)), ([BmimCl]­[MEA] (1:4)), ([HmimCl]­[MEA] (1:4))–with EtOH were systematically measured over the temperature range from 288.15 to 323.15 K, while Δ_mix_ *H* were determined at 298.15 and 308.15 K. Negative *V* ^E^ values across the entire compositional range reflect pronounced volume shrinkage and enhanced molecular packing upon mixing. The temperature dependence of *V* ^E^ suggests that packing effects dominate over H-bonding interactions. A pronounced U-shaped trend in Δη, with a minimum near *x*_1_ ≈ 0.6, suggests a breakdown of the original H-bond network in the DESs upon the addition of EtOH. The transition of Δ_mix_ *H* from positive to negative values with increasing the DES content indicates a progressive strengthening of the heteromolecular interactions between DESs and EtOH. The distinct composition dependences observed across different alkyl chain lengths of the cations highlight the complex interplay between chain structures and liquid microstructure. Further studies, such as molecular dynamics simulations, are therefore required to elucidate these effects at the molecular level.

Compared with H_2_O, EtOH interacts with DES in a more moderate manner. The strong polarity and high dielectric constant of H_2_O effectively screen Coulombic interactions between ions and facilitate the formation of the new H-bonding interactions, resulting in significant restructuring of the DES H-bond network and strong intermolecular associations. EtOH, with its lower polarity, reduced dielectric constant, and larger molecular volume, induces comparatively weaker structural rearrangement within the DES matrix. Consequently, while H-bonding interactions play a dominant role in the (DES + H_2_O) systems, the relative contribution of molecular packing effects becomes more pronounced in the (DES + EtOH) systems. These differences highlight the crucial role of polarity, dielectric constant, molecular size and structural characteristics of the cosolvent in determining the interaction pattern and structural evolution of DES-based mixtures. Compared with the imidazolium cation, the [Ch]^+^ cation is relatively smaller in size, which facilitates the formation of stronger intermolecular interactions with MEA, thereby resulting in a more compact molecular packing structure for the ChCl-based DES. This study provides crucial thermodynamic data and theoretical insights into the interaction mechanisms between amine-based DESs and cosolvents, offering valuable guidance for their applications.

## Supplementary Material



## Notes

Estimation of ideal liquidus lines; ^1^H NMR, ^13^C NMR, FTIR, and DSC spectra of the synthesized compounds; the ideal liquidus lines of the (EmimCl + MEA) and (BmimCl + MEA) mixtures; fitting parameters for the density and viscosity of EtOH; excess volumes, viscosity deviations, excess partial molar volumes and excess molar Gibbs energy of activation as well as the corresponding RK equation parameters for the studied (DES + EtOH) systems; and finally the enthalpies of mixing and the corresponding RK equation parameters for these systems (PDF)
