# Supplementary material for: Thermodynamic Study of Amine-Based Deep Eutectic Solvents with EtOH
Source: J Chem Eng Data. 2026 Jul 13;71(8):3381–93. doi: 10.1021/acs.jced.6c00157 (PMC13485107; doi:10.1021/acs.jced.6c00157)
Supplement: Supplementary file 1 [file je6c00157_si_001.pdf]

Supporting Information

Thermodynamic Study of Amine-based Deep Eutectic Solvents with EtOH

Fei Lin[a,b], Yusi Shen[a], Zhida Zuo[a,b], Linghong Lu[a], Xiaohua Lu[a,c], Yudan Zhu[a,c*], Xiaoyan Ji[b*]

[a]State Key Laboratory of Materials-Oriented Chemical Engineering, Nanjing Tech University, Nanjing 210009, P. R. China

[b]Division of Energy Science/Energy Engineering, Luleå University of Technology, 97187 Luleå, Sweden

[c]Suzhou Laboratory, Suzhou 215125, P.R. China

* Corresponding authors. E-mails: Yudan Zhu, ydzhu@njtech.edu.cn; Xiaoyan Ji, xiaoyan.ji@ltu.se

Contents

Supplementary Notes 3

Supplementary Figures 5

Supplementary Tables 11

References 26

Supplementary Notes

Estimation of ideal liquidus lines. Neglecting the small differences in heat capacities, the liquidus curve ($T_i(x_i)$) for an ideal binary liquid mixture ($i + j$) in equilibrium with pure component (i) should follow Equation 1

$$T_i = \frac{\Delta H_i^*}{\Delta H_i^* / T_i^* - R\,ln\,x_i} \tag{1}$$

where ΔH_i^* and T_i^* denote the enthalpy of fusion and the melting point of the pure component (IL or MEA), respectively, and R is the universal gas constant (R = 8.314 J·mol^{-1}·K^{-1}).

The reported melting points of MEA, [EmimCl], and [BmimCl] are 285.50,[1] 360.7,[2] and 341.48 K,[2] respectively, and their enthalpies of fusion are 13.8,[3] 15.35,[2] and 25.54 kJ·mol^{-1}.[2] Subsequently, the ideal liquidus lines were calculated using Equation 1. The results are shown in Figure S7.

Estimation of the uncertainty. The uncertainty mainly arises from (1) repeatability, (2) instrument accuracy and (3) temperature uncertainty.

(1) Repeatability can be assessed by averaging multiple measurements; according to the Bessel formula, the standard deviation s is expressed as

$$s = \sqrt{\sum \frac{(m_i - \bar{m})^2}{n-1}} \tag{2}$$

where n is the number of measurements, and thus the uncertainty of repeatability u_1 is expressed as

$$u_1 = \frac{s}{\sqrt{n}} \tag{3}$$

The repeatability of density is estimated as 1×10^{-6} g/cm^3. Alternatively, repeatability can be evaluated using the manufacturer-reported value r (e.g., 0.1% for viscosity), For a measured viscosity d, the repeatability uncertainty is given by $u_1 = r \cdot d$.

(2) Instrument uncertainty:

The accuracy of density/viscosity meter is d_1, and the resulting uncertainty can be evenly distributed ($k = \sqrt{3}$), $u_{2,1} = d_1 / k$ .The uncertainty is caused by resolution, $u_{2,2} = d_1 / 2k$. The synthetic uncertainty can be calculated by $u_2 = \sqrt{u_{2,1}^2 + u_{2,2}^2}$.

(3) Temperature uncertainty:

The relationship between density or viscosity data and temperature is determined using the linear regression, denoted as s_1. The temperature accuracy of density/viscosity meter is T_1(K). The uncertainty caused by resolution is $u_T = T_1/2k$. According to the equation, the temperature uncertainty can be calculated by $u_3 = s_1 \cdot u_T$.

The total uncertainty can be calculated as $u_\rho = \sqrt{u_1^2 + u_2^2 + u_3^2}$.

The expended uncertainty can be calculated as: taking the confidence probability as 95% and k = 2, the final extended uncertainty of density/viscosity is $\sigma_{(\rho,\eta)} = 2u_{(\rho,\eta)}$.

The uncertainties of derived parameters (e.g. V^E and $\Delta\eta$) were estimated using propagation of standard uncertainties based on partial derivatives of the measured variables.[4]

Supplementary Figures

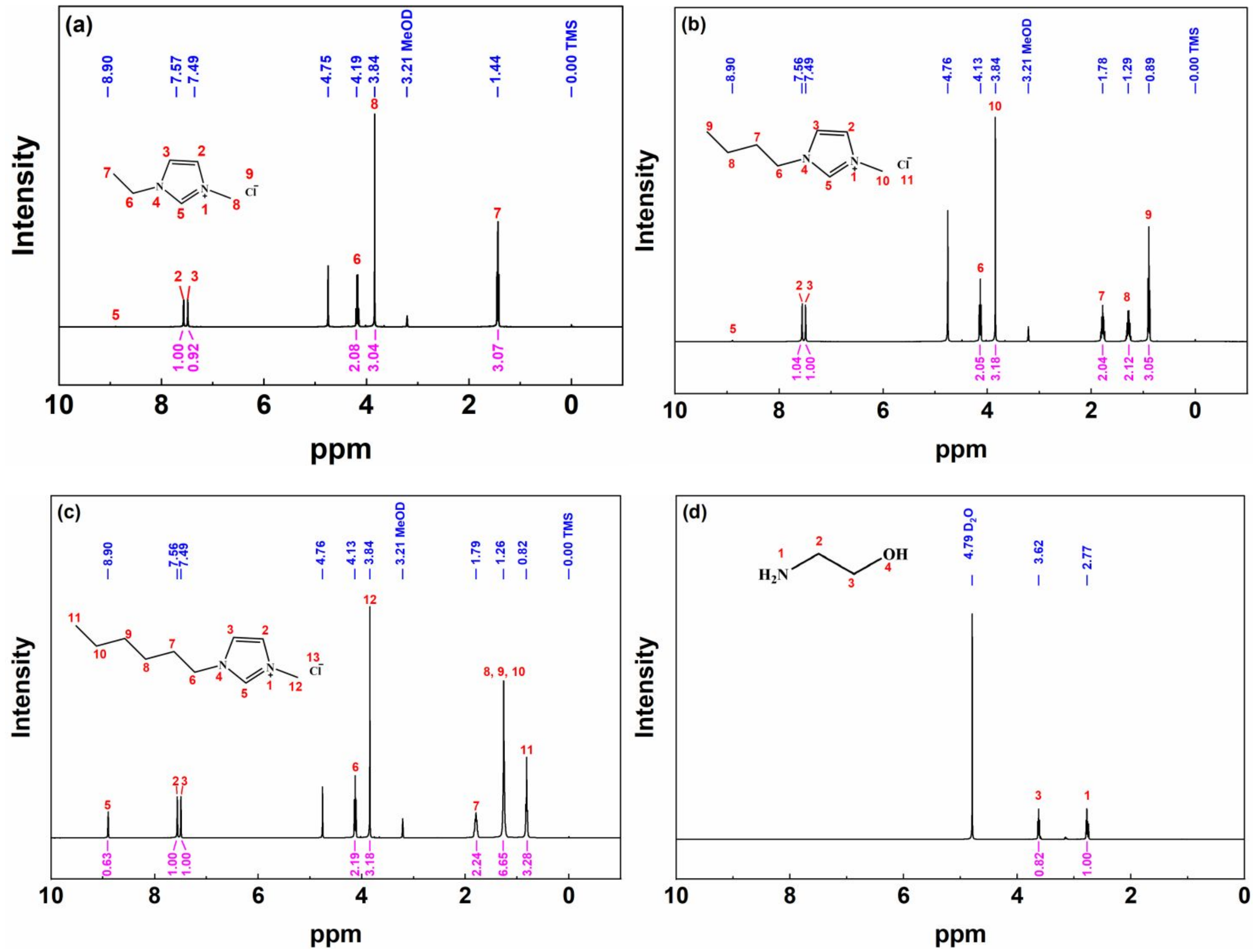

Figure S1. ^{1}H-NMR spectra of [EmimCl], [BmimCl], [HmimCl] and MEA.

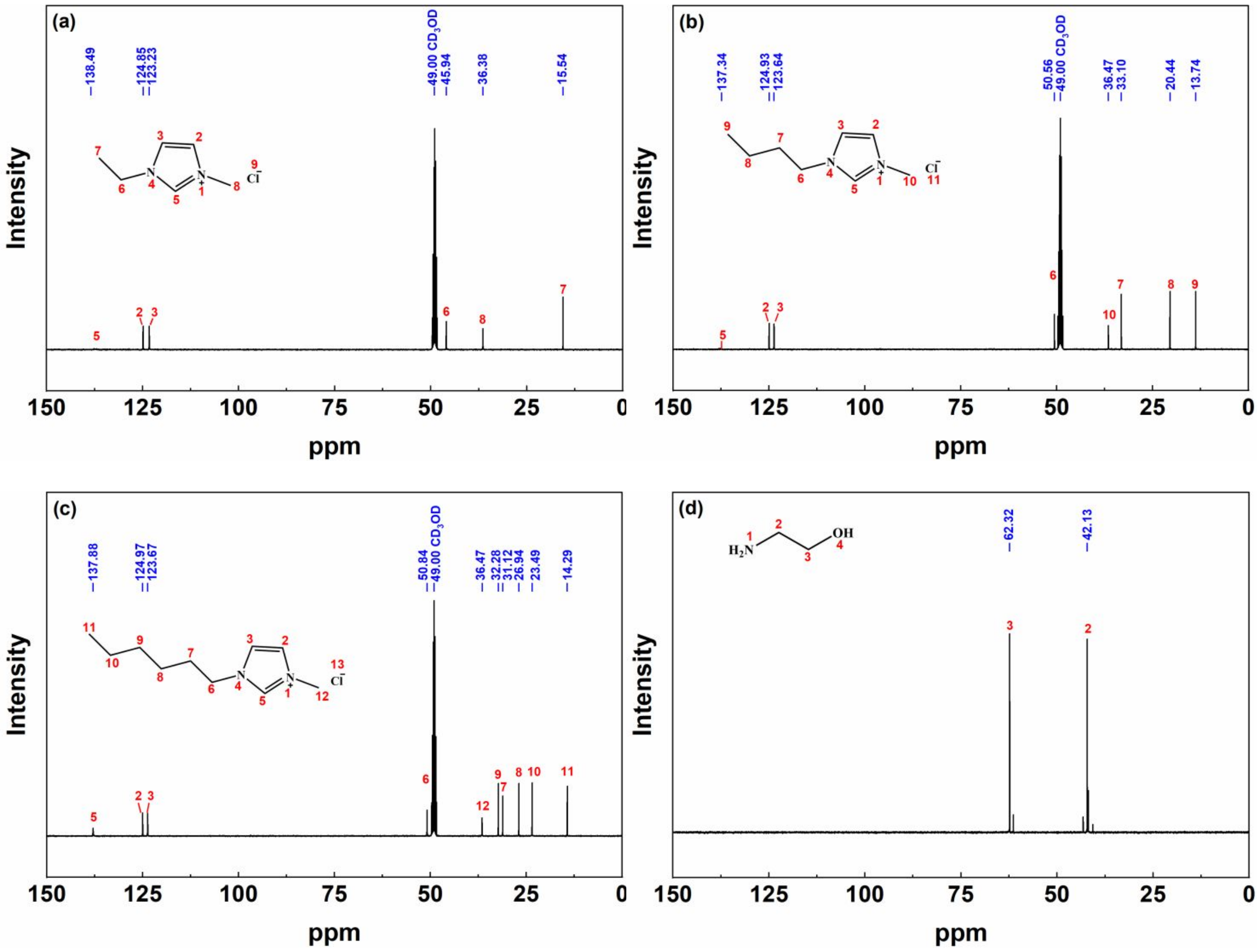

Figure S2. ^{13}C-NMR spectra of [EmimCl], [BmimCl], [HmimCl] and MEA.

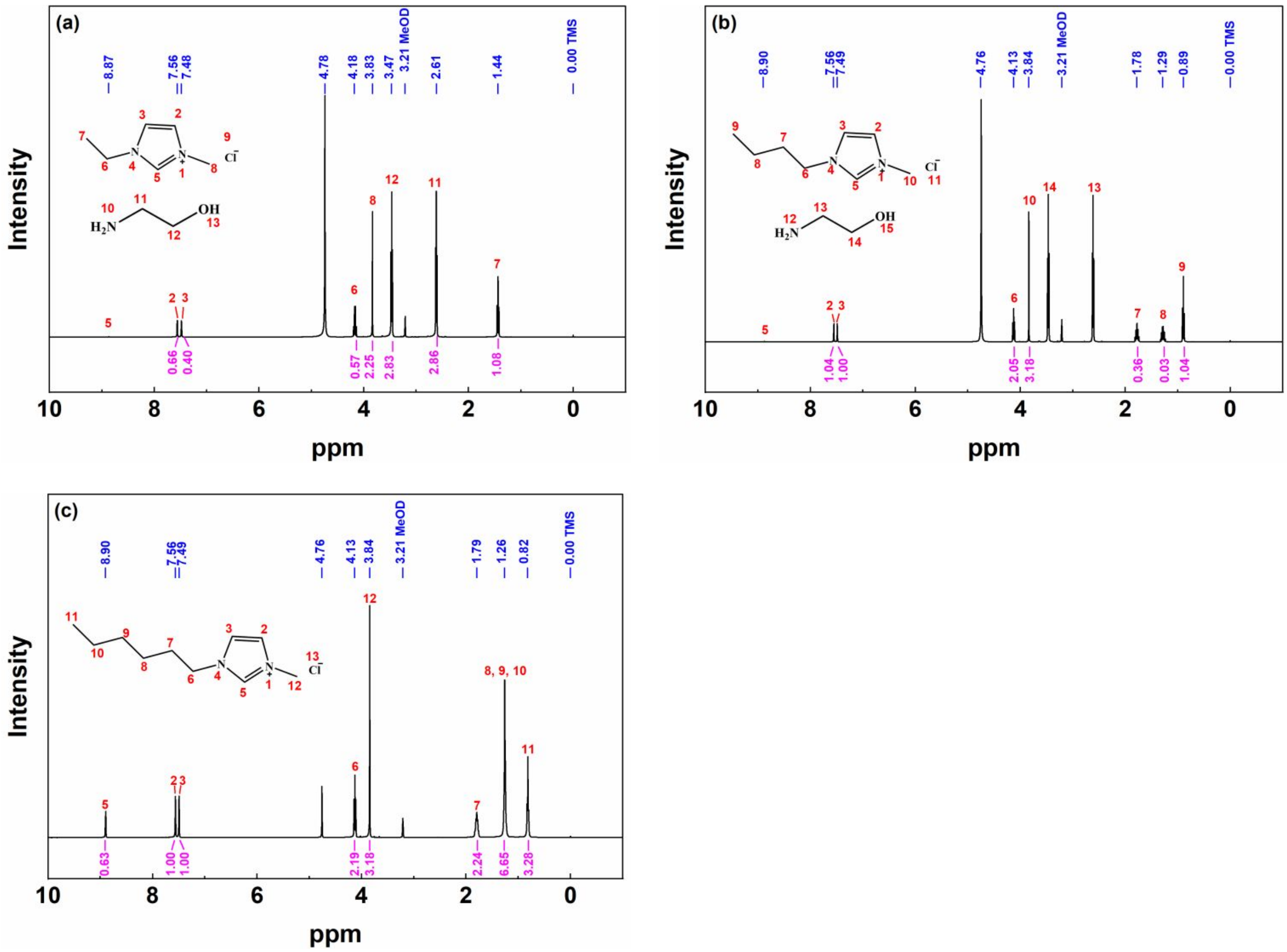

Figure S3. ^{1}H-NMR spectra of the ([EmimCl][MEA] (1:4)), ([BmimCl][MEA] (1:4)), and ([HmimCl][MEA] (1:4)).

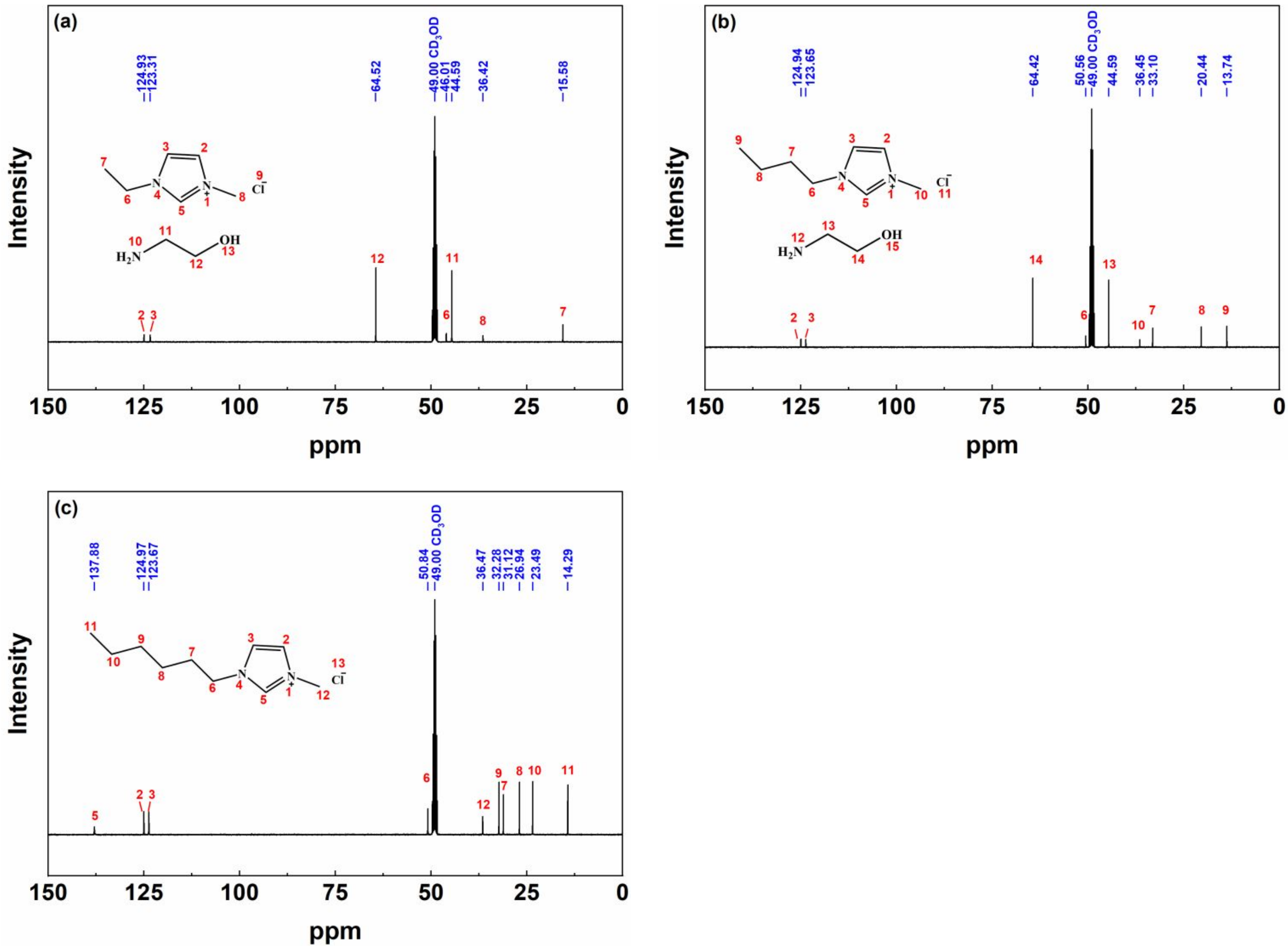

Figure S4. ^{13}C-NMR spectra of the ([EmimCl][MEA] (1:4)), ([BmimCl][MEA] (1:4)), and ([HmimCl][MEA] (1:4)).

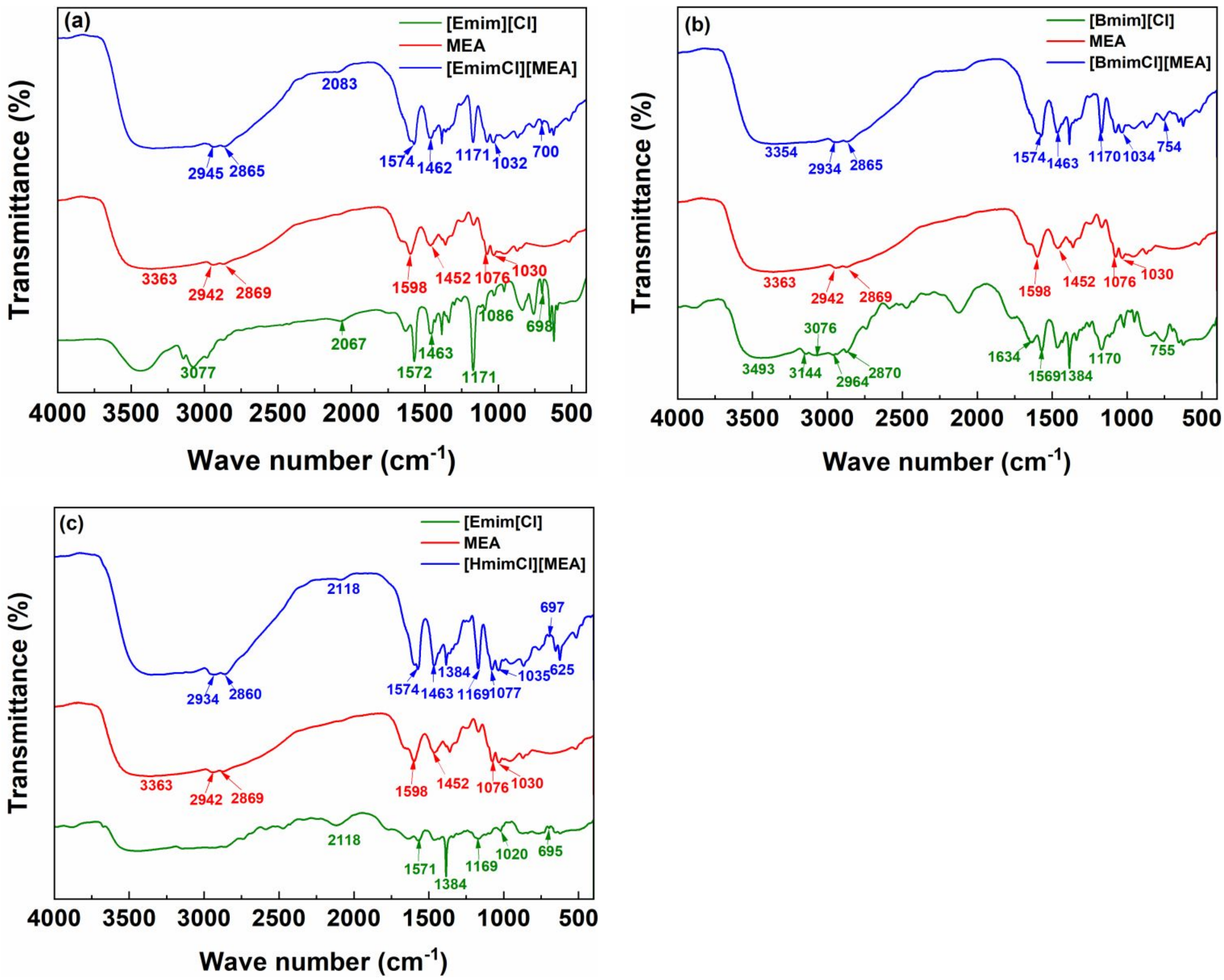

Figure S5. Comparison of FTIR spectra for the ([EmimCl][MEA] (1:4)), ([BmimCl][MEA] (1:4)), and ([HmimCl][MEA] (1:4)) and their pure components.

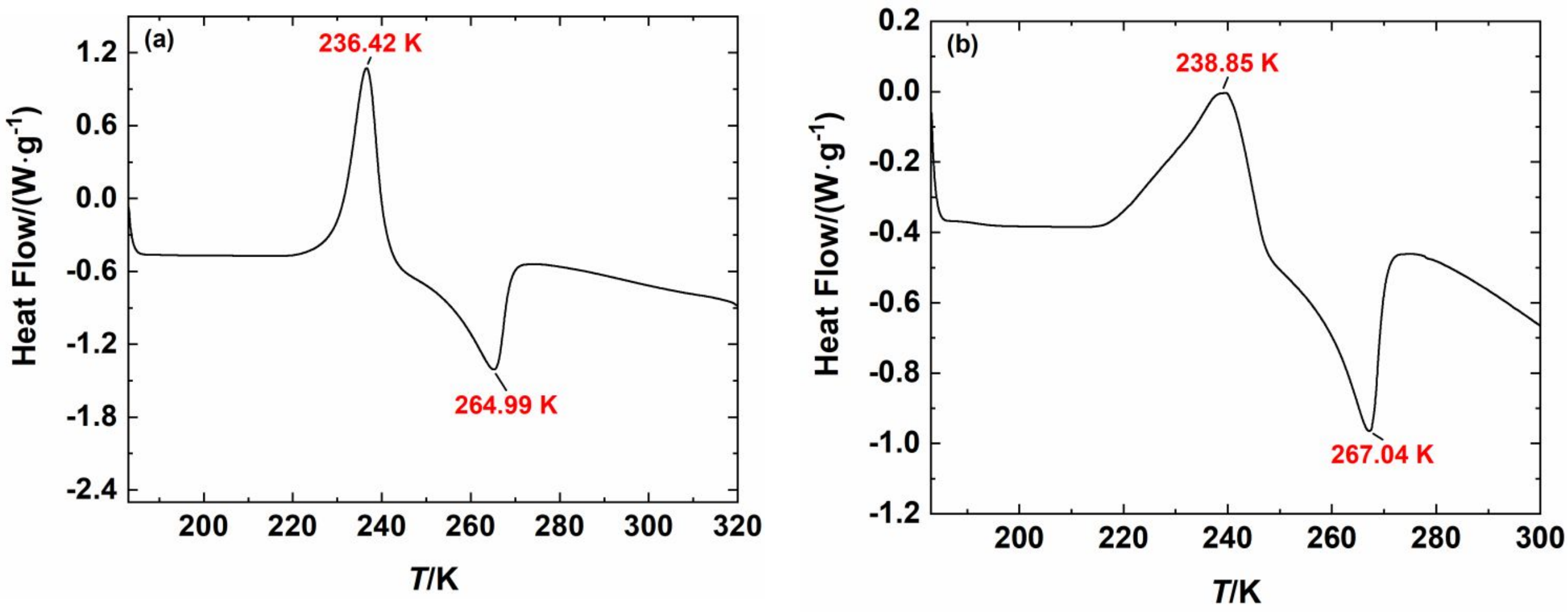

Figure S6. DSC traces of ([EmimCl][MEA] (1:4)) and ([BmimCl][MEA] (1:4)).[5]

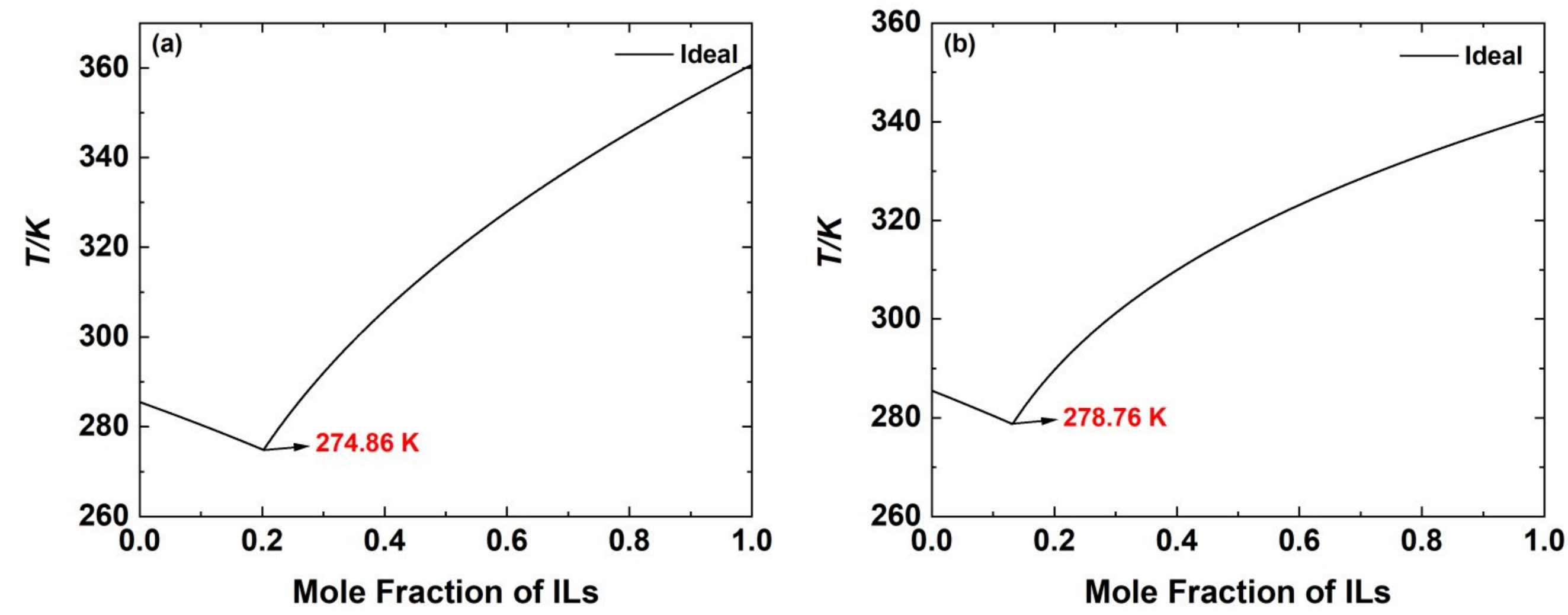

Figure S7. The ideal liquidus lines of the (EmimCl + MEA) and (BmimCl + MEA) mixtures calculated using Equation 1.

Supplementary Tables

Table S1. Parameters of the equation $\rho_{fit} = a_\rho + b_\rho T$ and the corresponding ARDs for fitting the density (ρ) of EtOH at 101 kPa.

Substances	a_ρ(g·cm^{-3})	b_ρ (g·cm^{-3}·K^{-1})	ARD[a] (%)
EtOH	1.0448	-0.000871	0.009

[a]Average relative deviation $ARD = 100\%/N\sum_{i=1}^{N}\left|(\rho_{cal}-\rho_{exp})/\rho_{exp}\right|$, where ρ_{cal}, ρ_{exp}, and N represent the calculated density, experimental density, and number of data points, respectively.

Table S2. Parameters of the equation $\ln(\eta_{fit}/\eta_0) = a_\eta + b_\eta/(c_\eta + T)$ (η_0 = 1 mPa·s)and the corresponding ARDs for fitting the logarithmic viscosity (η) of EtOH at 101 kPa.

Substances	a_η	b_η (K)	c_η (K)	ARD[a] (%)
EtOH	-5.5857	1749.65	10.58	0.221

[a]Average relative deviation $ARD = 100\%/N\sum_{i=1}^{N}\left|(\eta_{cal}-\eta_{exp})/\eta_{exp}\right|$ where η_{cal}, η_{exp}, and N represent the calculated density, experimental density, and number of data points, respectively.

Table S3. Enthalpies of mixing ($\Delta_{mix}H$) of EtOH (1) + H_2O (2) at 298.15 K and 101 kPa.[a]

x_1	$u(x_1)$	$\Delta_{mix}H$ (J·mol^{-1})	$u(\Delta_{mix}H)$
0.054	0.0002	-469.8	30.68
0.073	0.0004	-579.1	37.89
0.109	0.0006	-721.5	47.27
0.198	0.0011	-771.6	50.63
0.304	0.0010	-633.0	42.64
0.397	0.0013	-497.5	35.49
0.502	0.0018	-390.6	52.24

0.603	0.0019	-319.9	29.98
0.713	0.0009	-249.3	20.53
0.799	0.0028	-205.8	27.32
0.904	0.0002	-134.7	12.84

[a]Standard uncertainties u: $u(T)$ = 0.02 K, $u(p)$ = 3 kPa

Table S4. Excess molar volumes (V^E) of ([EmimCl][MEA] (1:4) + EtOH), ([BmimCl][MEA] (1:4) + EtOH), and ([HmimCl][MEA] (1:4) + EtOH) at different mole fractions (x_1) and temperatures (T) at 101 kPa.[a]

x_1	T (K)							
	288.15	293.15	298.15	303.15	308.15	313.15	318.15	323.15
	V^E (cm^3·mol^{-1}) of [EmimCl][MEA] (1:4) (1) + EtOH (2)							
0.000	0.0000	0.0000	0.0000	0.0000	0.0000	0.0000	0.0000	0.0000
0.100	-0.4093	-0.4232	-0.4384	-0.4539	-0.4703	-0.4881	-0.5063	-0.5259
0.205	-0.6152	-0.6356	-0.6577	-0.6800	-0.7039	-0.7296	-0.7564	-0.7857
0.302	-0.7402	-0.7646	-0.7892	-0.8152	-0.8418	-0.8714	-0.9011	-0.9344
0.401	-0.7804	-0.8047	-0.8300	-0.8558	-0.8835	-0.9129	-0.9436	-0.9758
0.500	-0.7680	-0.7904	-0.8141	-0.8387	-0.8643	-0.8924	-0.9208	-0.9515
0.600	-0.6647	-0.6841	-0.7042	-0.7249	-0.7470	-0.7703	-0.7950	-0.8213
0.704	-0.5502	-0.5652	-0.5804	-0.5967	-0.6139	-0.6323	-0.6519	-0.6723
0.800	-0.3799	-0.3910	-0.4012	-0.4120	-0.4242	-0.4363	-0.4506	-0.4642
0.896	-0.1995	-0.2051	-0.2106	-0.2158	-0.2220	-0.2282	-0.2355	-0.2428
1.000	0.0000	0.0000	0.0000	0.0000	0.0000	0.0000	0.0000	0.0000
	V^E (cm^3·mol^{-1}) of [BmimCl][MEA] (1:4) (1) + EtOH (2)							
0.000	0.0000	0.0000	0.0000	0.0000	0.0000	0.0000	0.0000	0.0000
0.103	-0.3885	-0.4025	-0.4173	-0.4329	-0.4487	-0.4667	-0.4849	-0.5046
0.201	-0.5917	-0.6122	-0.6333	-0.6559	-0.6785	-0.7047	-0.7308	-0.7598

0.302	-0.7089	-0.7319	-0.7561	-0.7816	-0.8083	-0.8368	-0.8663	-0.8984
0.397	-0.7454	-0.7692	-0.7938	-0.8189	-0.8456	-0.8744	-0.9047	-0.9371
0.501	-0.7240	-0.7451	-0.7681	-0.7919	-0.8171	-0.8439	-0.8725	-0.9026
0.574	-0.6813	-0.7002	-0.7215	-0.7435	-0.7665	-0.7909	-0.8167	-0.8443
0.686	-0.5617	-0.5776	-0.5941	-0.6108	-0.6288	-0.6492	-0.6697	-0.6921
0.800	-0.3948	-0.4048	-0.4157	-0.4273	-0.4396	-0.4531	-0.4667	-0.4824
0.906	-0.2018	-0.2039	-0.2070	-0.2112	-0.2181	-0.2271	-0.2366	-0.2467
1.000	0.0000	0.0000	0.0000	0.0000	0.0000	0.0000	0.0000	0.0000
V^E ($cm^3 \cdot mol^{-1}$) of [HmimCl][MEA] (1:4) (1) + EtOH (2)								
0.000	0.0000	0.0000	0.0000	0.0000	0.0000	0.0000	0.0000	0.0000
0.097	-0.3511	-0.3637	-0.3763	-0.3898	-0.4042	-0.4192	-0.4352	-0.4527
0.199	-0.5808	-0.5994	-0.6193	-0.6400	-0.6616	-0.6856	-0.7099	-0.7362
0.301	-0.6953	-0.7170	-0.7397	-0.7631	-0.7879	-0.8139	-0.8415	-0.8715
0.399	-0.7410	-0.7631	-0.7867	-0.8107	-0.8358	-0.8625	-0.8904	-0.9211
0.497	-0.7295	-0.7496	-0.7716	-0.7944	-0.8182	-0.8437	-0.8701	-0.8988
0.601	-0.6544	-0.6721	-0.6913	-0.7110	-0.7320	-0.7534	-0.7761	-0.8013
0.698	-0.5608	-0.5758	-0.5906	-0.6063	-0.6231	-0.6411	-0.6593	-0.6802
0.801	-0.4275	-0.4378	-0.4488	-0.4597	-0.4721	-0.4843	-0.4972	-0.5121
0.894	-0.2817	-0.2881	-0.2941	-0.3005	-0.3081	-0.3148	-0.3226	-0.3312
1.000	0.0000	0.0000	0.0000	0.0000	0.0000	0.0000	0.0000	0.0000

[a]Standard uncertainties are $u(x_1) = 0.004$, $u(T) = 0.01$ K, $u(p) = 3$ kPa. The combined expanded uncertainty is $U_c(V^E) = 0.047$ $cm^3 \cdot mol^{-1}$ (0.95 level of confidence).

Table S5. Parameters of excess molar volumes (V^E) fitted by RK equation and their ARDs for ([EmimCl][MEA] (1:4) + EtOH), ([BmimCl][MEA] (1:4) + EtOH), and ([HmimCl][MEA] (1:4) + EtOH) at different temperatures (T) at 101 kPa.

T (K)	A_1	A_2	A_3	A_4	A_5	ARD (%)
[EmimCl][MEA] (1:4) (1) + EtOH (2)						
288.15	-3.012	1.153	-0.289	0.068	-0.029	0.004

293.15	-3.102	1.203	-0.312	0.073	-0.031	0.005
298.15	-3.195	1.260	-0.333	0.079	-0.033	0.005
303.15	-3.292	1.316	-0.351	0.084	-0.036	0.005
308.15	-3.393	1.373	-0.378	0.091	-0.038	0.005
313.15	-3.503	1.439	-0.403	0.097	-0.041	0.006
318.15	-3.616	1.500	-0.438	0.105	-0.045	0.006
323.15	-3.737	1.574	-0.473	0.113	-0.049	0.006
[BmimCl][MEA] (1:4) (1) + EtOH (2)						
288.15	-2.894	0.993	-0.540	0.023	-0.035	0.006
293.15	-2.981	1.045	-0.557	0.027	-0.037	0.006
298.15	-3.075	1.096	-0.571	0.032	-0.039	0.007
303.15	-3.170	1.151	-0.598	0.037	-0.042	0.007
308.15	-3.270	1.203	-0.628	0.043	-0.045	0.007
313.15	-3.375	1.260	-0.682	0.048	-0.050	0.008
318.15	-3.486	1.319	-0.724	0.055	-0.054	0.008
323.15	-3.605	1.382	-0.783	0.061	-0.059	0.009
[HmimCl][MEA] (1:4) (1) + EtOH (2)						
288.15	-2.870	0.802	-0.803	-0.006	-0.047	0.008
293.15	-2.951	0.844	-0.834	-0.001	-0.050	0.008
298.15	-3.038	0.890	-0.857	0.004	-0.053	0.009
303.15	-3.128	0.936	-0.882	0.010	-0.056	0.009
308.15	-3.222	0.982	-0.916	0.015	-0.060	0.009
313.15	-3.322	1.037	-0.949	0.022	-0.063	0.010
318.15	-3.425	1.092	-0.984	0.028	-0.068	0.010
323.15	-3.540	1.148	-1.026	0.036	-0.072	0.011

[a]Average relative deviation $ARD = 100\%/N\sum_{i=1}^{N}\left|\left(V_{i,cal}^{E}-V_{i,exp}^{E}\right)/V_{i,exp}^{E}\right|$,where $V^{E}_{i,cal}$, $V^{E}_{i,exp}$, and N are the calculated and experimental excess molar volume, and the number of data, respectively.

Table S6. Excess partial molar volumes $\overline{V}_i^{\mathrm{E}}$ of pure component *i* in the mixture of ([EmimCl][MEA] (1:4) (1) + EtOH (2))[a]

x_1	*T* (K)							
	288.15	293.15	298.15	303.15	308.15	313.15	318.15	323.15
	$\overline{V}_1^{\mathrm{E}}$ ($cm^3 \cdot mol^{-1}$) of [EmimCl][MEA] (1:4) (1) + EtOH (2)							
0.000	-4.552	-4.722	-4.900	-5.079	-5.274	-5.484	-5.704	-5.946
0.100	-3.079	-3.182	-3.291	-3.402	-3.519	-3.647	-3.778	-3.921
0.205	-1.995	-2.055	-2.117	-2.181	-2.249	-2.322	-2.396	-2.476
0.302	-1.296	-1.330	-1.366	-1.403	-1.442	-1.484	-1.526	-1.571
0.401	-0.798	-0.817	-0.835	-0.856	-0.877	-0.899	-0.923	-0.946
0.500	-0.465	-0.475	-0.484	-0.494	-0.505	-0.516	-0.529	-0.541
0.600	-0.245	-0.250	-0.254	-0.258	-0.264	-0.269	-0.276	-0.281
0.704	-0.110	-0.112	-0.113	-0.115	-0.117	-0.119	-0.123	-0.125
0.800	-0.042	-0.043	-0.043	-0.043	-0.045	-0.045	-0.047	-0.048
0.896	-0.010	-0.010	-0.010	-0.010	-0.010	-0.011	-0.011	-0.012
1.000	0.000	0.000	0.000	0.000	0.000	0.000	0.000	0.000
	$\overline{V}_2^{\mathrm{E}}$ ($cm^3 \cdot mol^{-1}$) of [EmimCl][MEA] (1:4) (1) + EtOH (2)							
0.000	0.000	0.000	0.000	0.000	0.000	0.000	0.000	0.000
0.100	-0.075	-0.078	-0.081	-0.085	-0.089	-0.093	-0.097	-0.102
0.205	-0.267	-0.278	-0.289	-0.301	-0.314	-0.327	-0.342	-0.358
0.302	-0.502	-0.522	-0.542	-0.563	-0.585	-0.609	-0.634	-0.662
0.401	-0.770	-0.798	-0.828	-0.858	-0.889	-0.924	-0.959	-0.998
0.500	-1.041	-1.076	-1.114	-1.152	-1.192	-1.236	-1.279	-1.328
0.600	-1.308	-1.349	-1.392	-1.438	-1.484	-1.535	-1.586	-1.642
0.704	-1.559	-1.605	-1.654	-1.704	-1.756	-1.813	-1.870	-1.932
0.800	-1.764	-1.814	-1.865	-1.918	-1.974	-2.035	-2.097	-2.163
0.896	-1.940	-1.994	-2.046	-2.101	-2.162	-2.225	-2.295	-2.365
1.000	-2.109	-2.169	-2.223	-2.278	-2.346	-2.411	-2.495	-2.572

[a]Standard uncertainties are $u(x_1) = 0.004$, $u(T) = 0.01$ K, $u(p) = 3$ kPa. The standard uncertainty $u(\overline{V}_i^{\mathrm{E}}) \approx 0.084\ cm^3 \cdot mol^{-1}$.

Table S7. Excess partial molar volumes $\overline{V}_i^{\mathrm{E}}$ of pure component *i* in the mixture of ([BmimCl][MEA] (1:4) (1) + EtOH (2))[a]

x_1	T (K)							
	288.15	293.15	298.15	303.15	308.15	313.15	318.15	323.15
	$\overline{V}_1^E$ (cm^3·mol^{-1}) of [BmimCl][MEA] (1:4) (1) + EtOH (2)							
0.000	-4.484	-4.648	-4.814	-4.998	-5.188	-5.415	-5.638	-5.890
0.103	-2.934	-3.034	-3.137	-3.247	-3.359	-3.485	-3.613	-3.754
0.201	-1.921	-1.981	-2.045	-2.110	-2.176	-2.245	-2.319	-2.396
0.302	-1.222	-1.256	-1.294	-1.330	-1.368	-1.404	-1.445	-1.486
0.397	-0.783	-0.802	-0.823	-0.843	-0.865	-0.885	-0.908	-0.932
0.501	-0.472	-0.481	-0.491	-0.501	-0.513	-0.525	-0.538	-0.552
0.574	-0.327	-0.332	-0.338	-0.344	-0.352	-0.361	-0.370	-0.381
0.686	-0.176	-0.177	-0.179	-0.182	-0.186	-0.192	-0.198	-0.205
0.800	-0.078	-0.078	-0.078	-0.080	-0.082	-0.085	-0.088	-0.093
0.906	-0.020	-0.020	-0.020	-0.020	-0.021	-0.022	-0.023	-0.025
1.000	0.000	0.000	0.000	0.000	0.000	0.000	0.000	0.000
	$\overline{V}_2^E$ (cm^3·mol^{-1}) of [BmimCl][MEA] (1:4) (1) + EtOH (2)							
0.000	0.000	0.000	0.000	0.000	0.000	0.000	0.000	0.000
0.103	-0.080	-0.083	-0.086	-0.090	-0.094	-0.099	-0.104	-0.110
0.201	-0.258	-0.269	-0.279	-0.290	-0.302	-0.317	-0.332	-0.348
0.302	-0.490	-0.509	-0.528	-0.549	-0.570	-0.596	-0.621	-0.650
0.397	-0.724	-0.751	-0.778	-0.808	-0.838	-0.872	-0.906	-0.944
0.501	-0.975	-1.010	-1.046	-1.084	-1.122	-1.163	-1.205	-1.251
0.574	-1.142	-1.182	-1.224	-1.266	-1.308	-1.353	-1.400	-1.449
0.686	-1.399	-1.444	-1.492	-1.540	-1.589	-1.637	-1.691	-1.745
0.800	-1.682	-1.730	-1.783	-1.835	-1.890	-1.946	-2.007	-2.071
0.906	-2.023	-2.073	-2.127	-2.184	-2.247	-2.320	-2.392	-2.474
1.000	-2.453	-2.503	-2.557	-2.621	-2.697	-2.798	-2.890	-3.004

[a]Standard uncertainties are $u(x_1) = 0.004$, $u(T) = 0.01$ K, $u(p) = 3$ kPa. The standard uncertainty $u(\overline{V}_i^E) \approx$ 0.074 cm^3·mol^{-1}.

Table S8. Excess partial molar volumes $\overline{V}_i^E$ of pure component *i* in the mixture of ([HmimCl][MEA] (1:4) (1) + EtOH (2))[a]

x_1	T (K)							
	288.15	293.15	298.15	303.15	308.15	313.15	318.15	323.15

			$\overline{V}_1^{E}$ (cm^3·mol^{-1}) of [HmimCl][MEA] (1:4) (1) + EtOH (2)					
0.000	-4.517	-4.678	-4.841	-5.012	-5.195	-5.393	-5.598	-5.822
0.097	-2.962	-3.058	-3.159	-3.263	-3.373	-3.492	-3.614	-3.748
0.199	-1.878	-1.932	-1.991	-2.051	-2.114	-2.181	-2.250	-2.326
0.301	-1.194	-1.223	-1.257	-1.292	-1.327	-1.364	-1.403	-1.445
0.399	-0.781	-0.798	-0.817	-0.836	-0.857	-0.877	-0.899	-0.924
0.497	-0.524	-0.534	-0.544	-0.555	-0.568	-0.579	-0.591	-0.606
0.601	-0.346	-0.352	-0.357	-0.363	-0.370	-0.377	-0.384	-0.393
0.698	-0.225	-0.229	-0.232	-0.235	-0.240	-0.243	-0.247	-0.253
0.801	-0.120	-0.123	-0.124	-0.125	-0.128	-0.130	-0.132	-0.135
0.894	-0.042	-0.043	-0.044	-0.044	-0.045	-0.046	-0.047	-0.048
1.000	0.000	0.000	0.000	0.000	0.000	0.000	0.000	0.000
			$\overline{V}_2^{E}$ (cm^3·mol^{-1}) of [HmimCl][MEA] (1:4) (1) + EtOH (2)					
0.000	0.000	0.000	0.000	0.000	0.000	0.000	0.000	0.000
0.097	-0.075	-0.078	-0.081	-0.084	-0.088	-0.092	-0.096	-0.100
0.199	-0.260	-0.270	-0.280	-0.290	-0.302	-0.314	-0.327	-0.341
0.301	-0.485	-0.503	-0.521	-0.540	-0.561	-0.583	-0.606	-0.631
0.399	-0.705	-0.729	-0.756	-0.783	-0.811	-0.842	-0.874	-0.909
0.497	-0.911	-0.942	-0.975	-1.009	-1.043	-1.082	-1.121	-1.164
0.601	-1.127	-1.162	-1.201	-1.241	-1.281	-1.326	-1.372	-1.421
0.698	-1.351	-1.390	-1.434	-1.479	-1.524	-1.574	-1.625	-1.680
0.801	-1.667	-1.711	-1.760	-1.810	-1.862	-1.916	-1.973	-2.037
0.894	-2.108	-2.160	-2.214	-2.269	-2.330	-2.392	-2.457	-2.531
1.000	-2.924	-2.992	-3.054	-3.120	-3.200	-3.276	-3.357	-3.453

[a]Standard uncertainties are $u(x_1)$ = 0.004, $u(T)$ = 0.01 K, $u(p)$ = 3 kPa. The standard uncertainty $u(\overline{V}_i^{E})$ ≈ 0.079 cm^3·mol^{-1}.

Table S9. Viscosity deviations ($\Delta\eta$) of ([EmimCl][MEA] (1:4) + EtOH), ([BmimCl][MEA] (1:4) + EtOH), and ([HmimCl][MEA] (1:4) + EtOH) at different temperatures (T) and mole fractions (x_1) at 101 kPa.[a]

x_1	T (K)							
	288.15	293.15	298.15	303.15	308.15	313.15	318.15	323.15
			$\Delta\eta$ (mPa·s) of [EmimCl][MEA] (1:4) (1) + EtOH (2)					

0.000	0.00	0.00	0.00	0.00	0.00	0.00	0.00	0.00
0.100	-2.89	-2.16	-1.63	-1.25	-0.97	-0.77	-0.62	-0.51
0.205	-5.73	-4.26	-3.19	-2.44	-1.89	-1.49	-1.20	-0.98
0.302	-7.98	-5.93	-4.42	-3.37	-2.60	-2.05	-1.65	-1.34
0.401	-9.79	-7.22	-5.37	-4.06	-3.11	-2.43	-1.94	-1.57
0.500	-11.05	-8.10	-5.95	-4.47	-3.40	-2.65	-2.11	-1.70
0.600	-11.83	-8.65	-6.32	-4.75	-3.61	-2.80	-2.22	-1.79
0.704	-11.45	-8.37	-6.08	-4.54	-3.42	-2.66	-2.12	-1.71
0.800	-9.56	-6.92	-4.93	-3.66	-2.74	-2.11	-1.69	-1.37
0.896	-5.78	-4.22	-2.94	-2.15	-1.58	-1.22	-0.98	-0.81
1.000	0.00	0.00	0.00	0.00	0.00	0.00	0.00	0.00
$\Delta\eta$ (mPa·s) of [BmimCl][MEA] (1:4) (1)+ EtOH (2)								
0.000	0.00	0.00	0.00	0.00	0.00	0.00	0.00	0.00
0.103	-4.21	-3.09	-2.33	-1.78	-1.38	-1.09	-0.86	-0.70
0.201	-8.03	-5.89	-4.42	-3.37	-2.61	-2.06	-1.63	-1.31
0.302	-11.50	-8.42	-6.30	-4.80	-3.71	-2.91	-2.31	-1.86
0.397	-14.27	-10.39	-7.73	-5.86	-4.51	-3.52	-2.78	-2.22
0.501	-16.41	-11.86	-8.77	-6.62	-5.07	-3.94	-3.09	-2.46
0.574	-17.17	-12.34	-9.09	-6.84	-5.21	-4.04	-3.16	-2.51
0.686	-16.76	-11.88	-8.68	-6.47	-4.91	-3.79	-2.96	-2.34
0.800	-14.70	-10.38	-7.58	-5.64	-4.25	-3.28	-2.55	-2.01
0.906	-10.39	-6.83	-4.95	-3.49	-2.52	-1.94	-1.51	-1.19
1.000	0.00	0.00	0.00	0.00	0.00	0.00	0.00	0.00
$\Delta\eta$ (mPa·s) of [HmimCl][MEA] (1:4) (1) + EtOH (2)								
0.000	0.00	0.00	0.00	0.00	0.00	0.00	0.00	0.00
0.097	-4.45	-3.22	-2.42	-1.84	-1.43	-1.12	-0.89	-0.72
0.199	-8.90	-6.43	-4.81	-3.66	-2.84	-2.22	-1.77	-1.42
0.301	-12.84	-9.23	-6.86	-5.20	-4.00	-3.12	-2.48	-1.98
0.399	-15.92	-11.36	-8.41	-6.34	-4.85	-3.77	-2.97	-2.37

0.497	-18.50	-13.20	-9.78	-7.38	-5.66	-4.37	-3.45	-2.76
0.601	-19.65	-13.99	-10.31	-7.76	-5.93	-4.60	-3.63	-2.86
0.698	-18.27	-12.52	-9.05	-6.68	-5.02	-3.81	-2.96	-2.32
0.801	-15.33	-10.31	-7.48	-5.50	-4.14	-3.15	-2.43	-1.90
0.894	-9.58	-6.25	-4.38	-3.10	-2.29	-1.72	-1.29	-0.99
1.000	0.00	0.00	0.00	0.00	0.00	0.00	0.00	0.00

[a]Standard uncertainties are $u(x_1) = 0.004$, $u(T) = 0.01$ K, $u(p) = 3$ kPa. The relative expanded uncertainty is $U_r(\Delta\eta) = 0.037$ (0.95 level of confidence).

Table S10. Parameters of viscosity deviations ($\Delta\eta$) fitted by RK equation and their ARDs for ([EmimCl][MEA] (1:4) + EtOH), ([BmimCl][MEA] (1:4) + EtOH), and ([HmimCl][MEA] (1:4) + EtOH) at different temperatures (T) at 101 kPa.

T (K)	A_1	A_2	A_3	A_4	A_5	ARD (%)
			[EmimCl][MEA] (1:4) (1) + EtOH (2)			
288.15	-45.008	-20.407	-7.445	-2.279	-0.503	0.002
293.15	-33.034	-14.214	-5.244	-1.758	-0.449	0.004
298.15	-24.353	-9.665	-3.040	-0.508	0.083	0.005
303.15	-18.351	-6.830	-2.098	-0.312	0.082	0.005
308.15	-14.006	-4.816	-1.405	-0.151	0.090	0.005
313.15	-10.917	-3.558	-1.048	-0.086	0.084	0.005
318.15	-8.691	-2.737	-0.968	-0.225	-0.003	0.001
323.15	-7.004	-2.135	-0.869	-0.266	-0.035	0.002
			[BmimCl][MEA] (1:4) (1) + EtOH (2)			
288.15	-63.959	-30.634	-12.590	-6.580	-3.104	0.030
293.15	-46.510	-20.886	-7.252	-2.121	-0.474	0.002
298.15	-34.419	-14.537	-4.886	-1.427	-0.314	0.002
303.15	-26.092	-10.455	-3.056	-0.273	0.246	0.009
308.15	-20.039	-7.593	-1.934	0.216	0.412	0.015
313.15	-15.576	-5.623	-1.484	0.076	0.252	0.012

318.15	-12.248	-4.195	-1.060	0.134	0.226	0.012
323.15	-9.761	-3.189	-0.747	0.208	0.224	0.013
[HmimCl][MEA] (1:4) (1) + EtOH (2)						
288.15	-73.621	-35.743	-7.592	8.144	7.623	0.081
293.15	-52.413	-23.491	-1.968	10.009	7.774	0.105
298.15	-38.752	-16.399	-0.343	8.116	5.892	0.102
303.15	-29.238	-11.709	0.696	6.953	4.737	0.103
308.15	-22.379	-8.474	1.044	5.541	3.572	0.096
313.15	-17.318	-6.134	1.157	4.324	2.631	0.086
318.15	-13.669	-4.588	1.286	3.698	2.137	0.085
323.15	-10.872	-3.372	1.279	3.009	1.642	0.076

[a]Average relative deviation $ARD = 100\%/N\sum_{i=1}^{N}\left|\left(\Delta\eta_{cal}-\Delta\eta_{exp}\right)/\Delta\eta_{exp}\right|$, where $\Delta\eta_{i,cal}$, $\Delta\eta_{i,exp}$, and N are the calculated and experimental viscosity deviation, and the number of data, respectively.

Table S11. Excess molar Gibbs energy of activation ($G^{*,E}$) of ([EmimCl][MEA] (1:4) + EtOH), ([BmimCl][MEA] (1:4) + EtOH), and ([HmimCl][MEA] (1:4) + EtOH) at different temperatures (T) and mole fractions (x_1) at 101 kPa.[a]

x_1	T (K)							
	288.15	293.15	298.15	303.15	308.15	313.15	318.15	323.15
$G^{*,E}$(J·mol^{-1}) of [EmimCl][MEA] (1:4) (1) + EtOH (2)								
0.000	0.00	0.00	0.00	0.00	0.00	0.00	0.00	0.00
0.100	140.16	163.25	184.50	203.14	218.15	234.07	242.99	251.60
0.205	232.83	270.17	302.17	332.80	358.64	384.18	401.17	416.84
0.302	278.05	320.92	356.79	392.89	424.81	454.20	475.91	495.45
0.402	291.89	334.28	371.20	408.41	443.12	472.67	495.65	516.16
0.500	279.84	317.08	354.00	388.82	423.28	450.28	470.84	489.41
0.601	247.16	276.02	312.51	342.29	373.81	396.36	411.33	425.50
0.704	197.24	215.98	250.94	273.70	299.75	316.62	323.97	332.14
0.800	140.50	150.31	181.11	196.50	215.63	226.87	227.57	230.33

0.896	76.32	79.33	100.51	108.34	119.02	124.70	121.70	120.92
1.000	0.00	0.00	0.00	0.00	0.00	0.00	0.00	0.00
$G^{\square,E}$(J·mol^{-1}) of [BmimCl][MEA] (1:4) (1) + EtOH (2)								
0.000	0.00	0.00	0.00	0.00	0.00	0.00	0.00	0.00
0.103	118.96	155.29	176.31	201.23	222.52	238.34	254.52	269.30
0.201	209.73	256.39	289.21	322.16	352.13	377.91	402.75	424.57
0.302	273.43	318.09	356.29	388.61	420.27	451.18	479.71	503.85
0.397	303.65	342.73	381.14	409.46	439.23	470.65	499.20	522.67
0.501	302.86	337.46	372.17	395.99	422.35	450.28	476.23	497.20
0.574	282.98	316.93	347.39	369.11	393.13	416.77	439.87	458.60
0.686	225.76	261.92	284.21	304.51	325.29	340.49	358.20	373.12
0.800	145.44	182.67	196.07	215.11	232.15	238.73	250.38	261.09
0.906	63.78	91.40	97.05	110.59	121.33	122.24	127.89	133.78
1.000	0.00	0.00	0.00	0.00	0.00	0.00	0.00	0.00
$G^{\square,E}$(J·mol^{-1}) of [HmimCl][MEA] (1:4) (1) + EtOH (2)								
0.000	0.00	0.00	0.00	0.00	0.00	0.00	0.00	0.00
0.097	135.38	178.68	202.86	226.82	244.74	260.28	276.65	285.47
0.199	208.99	266.42	301.71	335.18	363.19	390.44	414.38	431.39
0.301	242.57	297.54	334.13	367.37	398.56	432.29	457.47	480.39
0.399	258.25	306.10	338.59	367.18	396.83	432.07	455.17	481.08
0.497	268.64	311.35	337.45	360.40	385.63	418.24	438.02	464.02
0.601	276.67	318.90	337.83	355.71	374.57	400.79	416.93	440.05
0.698	274.69	319.89	333.03	347.73	360.27	378.47	391.60	409.77
0.801	246.03	292.31	300.71	312.92	319.53	328.93	339.07	350.62
0.894	173.27	210.22	215.00	223.83	226.35	229.26	235.92	241.30
1.000	0.00	0.00	0.00	0.00	0.00	0.00	0.00	0.00

[a]Standard uncertainties are $u(x_1)$ = 0.004, $u(T)$ = 0.01 K, $u(p)$ = 3 kPa. The combined expanded relative uncertainties is $U_r(G^{\square,E})$ = 0.82 (0.95 level of confidence).

Table S12. Enthalpies of mixing ($\Delta_{mix}H$) of ([EmimCl][MEA] (1:4) +EtOH), ([BmimCl][MEA] (1:4) + EtOH), and ([HmimCl][MEA] (1:4) + EtOH) at temperatures (T) and mole fractions (x_1) at 101 kPa.[a]

x_1	$u(x_1)$	$\Delta_{mix}H$ (J·mol^{-1})	$u(\Delta_{mix}H)$	x_1	$u(x_1)$	$\Delta_{mix}H$ (J·mol^{-1})	$u(\Delta_{mix}H)$	x_1	$u(x_1)$	$\Delta_{mix}H$ (J·mol^{-1})	$u(\Delta_{mix}H)$
[EmimCl][MEA] (1:4) (1) + EtOH (2)				[BmimCl][MEA] (1:4) (1) + EtOH (2)				[HmimCl][MEA] (1:4) (1) + EtOH (2)			
T = 298.15 K											
0.106	0.0001	10.6	4.42	0.074	0.0001	33.2	2.15	0.101	0.0002	41.0	3.14
0.199	0.0029	-21.5	8.09	0.118	0.0045	-9.8	13.51	0.197	0.0010	-10.8	8.11
0.302	0.0027	-103.0	7.00	0.150	0.0020	-63.9	9.79	0.293	0.0009	-49.7	4.22
0.387	0.0002	-199.6	17.06	0.247	0.0009	-241.5	20.38	0.399	0.0010	-325.7	22.33
0.520	0.0008	-206.8	13.69	0.304	0.0009	-288.9	23.26	0.494	0.0009	-356.7	25.91
0.611	0.0161	-224.7	18.85	0.407	0.0004	-360.7	25.30	0.600	0.0011	-351.6	23.84
0.700	0.0002	-200.1	17.05	0.492	0.0006	-376.5	25.16	0.698	0.0008	-303.2	22.27
0.794	0.0018	-122.7	15.05	0.602	0.0030	-357.9	23.10	0.797	0.0013	-223.6	17.06
0.907	0.0033	-55.1	3.57	0.699	0.0012	-298.9	19.62	0.894	0.0013	-126.4	8.55
				0.791	0.0063	-221.6	17.49				
				0.902	0.0016	-110.1	7.37				

					T = 308.15 K						
0.107	0.0005	44.0	8.21	0.056	0.0011	63.3	4.32	0.117	0.0154	39.8	11.67
0.275	0.0001	-77.6	5.87	0.115	0.0014	-18.1	1.73	0.146	0.0000	-11.7	6.16
0.402	0.0009	-180.4	19.80	0.151	0.0021	-37.6	7.71	0.204	0.0001	-37.2	14.86
0.506	0.0013	-187.4	17.60	0.249	0.0001	-175.3	11.35	0.295	0.0022	-73.0	25.52
0.595	0.0000	-193.5	12.95	0.307	0.0003	-202.5	13.44	0.399	0.0009	-262.1	21.98
0.702	0.0002	-154.1	10.23	0.408	0.0018	-276.1	25.50	0.496	0.0014	-279.8	21.57
0.797	0.0001	-110.6	8.82	0.492	0.0012	-276.8	28.04	0.595	0.0019	-266.5	17.54
0.904	0.0013	-55.4	3.58	0.600	0.0007	-272.6	23.24	0.698	0.0017	-207.3	31.40
				0.694	0.0012	-250.8	18.46	0.798	0.0004	-142.9	13.14
				0.794	0.0028	-184.5	26.80	0.893	0.0007	-79.5	9.44
				0.898	0.0008	-75.3	20.63				

[a]Standard uncertainties u: $u(T)$ = 0.02 K, $u(p)$ = 3 kPa

Table S13. Parameters of enthalpies of mixing ($\Delta_{mix}H$) fitted by RK equation and their coefficient of determination (R^2) for ([EmimCl][MEA] (1:4) + EtOH), ([BmimCl][MEA] (1:4) + EtOH), and ([HmimCl][MEA] (1:4) + EtOH) at different temperatures (T) at 101 kPa.[a]

T (K)	A_1	A_2	A_3	A_4	A_5	R^2
			[EmimCl][MEA] (1:4) (1) + EtOH (2)			
298.15	-899.196	-436.665	1238.072	-160.194	-279.339	0.9830
308.15	-788.483	-163.719	1065.918	-889.043	115.030	0.9964
			[BmimCl][MEA] (1:4) (1) + EtOH (2)			
298.15	-1503.172	219.500	270.942	-1607.886	1905.368	0.9981
308.15	-1118.621	-25.892	-296.349	-949.339	2466.588	0.9933
			[HmimCl][MEA] (1:4) (1) + EtOH (2)			
298.15	-1484.857	-1039.849	3689.801	-238.282	-3452.055	0.9697
308.15	-1148.875	-451.957	2692.679	-391.635	-2260.562	0.9711

[a] Coefficient of determination $R^2 = 1 - \sum \left(\Delta_{mix}H_{i,exp} - \Delta_{mix}H_{i,cal}\right)^2 / \sum \left(\Delta_{mix}H_{i,exp} - \Delta_{mix}H_{avg}\right)^2$, where $\Delta_{mix}H_{i,cal}$, $\Delta_{mix}H_{i,exp}$, and $\Delta_{mix}H_{avg}$ are the calculated, experimental , and the average enthalpies of mixing, respectively.

References

(1) Cao, L.; Huang, J.; Zhang, X.; Zhang, S.; Gao, J.; Zeng, S., Imidazole tailored deep eutectic solvents for CO_2 capture enhanced by hydrogen bonds. *Phys. Chem. Chem. Phys.* **2015,** *17*, 27306-27316.

(2) Kick, M.; Keil, P.; König, A., Solid–liquid phase diagram of the two Ionic Liquids EMIMCl and BMIMCl. *Fluid Phase Equilib.* **2013,** *338*, 172-178.

(3) Penttilä, A.; Uusi-Kyyny, P.; Salminen, A.; Seppälä, J.; Alopaeus, V., A comprehensive thermodynamic study of heat stable acetic acid salt of monoethanolamine. *International Journal of Greenhouse Gas Control* **2014,** *22*, 313-324.

(4) Berendsen, H. J. C., *A Student's Guide to Data and Error Analysis*. Cambridge University Press: Cambridge, 2011.

(5) Zuo, Z.; Shen, Y.; Lu, L.; Zhu, Y.; Lu, X.; Ji, X., Thermodynamic study of amine-based deep eutectic solvents with H_2O. *J. Chem. Eng. Data* **2026,** *359*, 130559.
